# Functional connectivity discriminates epileptogenic states and predicts surgical outcome in children with drug resistant epilepsy

**DOI:** 10.1038/s41598-023-36551-0

**Published:** 2023-06-14

**Authors:** Sakar Rijal, Ludovica Corona, M. Scott Perry, Eleonora Tamilia, Joseph R. Madsen, Scellig S. D. Stone, Jeffrey Bolton, Phillip L. Pearl, Christos Papadelis

**Affiliations:** 1 Jane and John Justin Institute for Mind Health Neurosciences Center, Cook Children’s Health Care System, 1500 Cooper St., Fort Worth, TX 76104 USA; 2 Department of Bioengineering, The University of Texas at Arlington, Arlington, TX 76010 USA; 3 Fetal-Neonatal Neuroimaging and Developmental Science Center, Boston Children’s Hospital, Harvard Medical School, Boston, MA 02115 USA; 4 Division of Epilepsy Surgery, Department of Neurosurgery, Boston Children’s Hospital, Harvard Medical School, Boston, MA 02115 USA; 5 Division of Epilepsy and Clinical Neurophysiology, Department of Neurology, Boston Children’s Hospital, Harvard Medical School, Boston, MA 02115 USA; 6 School of Medicine, Texas Christian University, Fort Worth, TX 76129 USA

**Keywords:** Epilepsy, Diagnostic markers, Predictive markers, Prognostic markers

## Abstract

Normal brain functioning emerges from a complex interplay among regions forming networks. In epilepsy, these networks are disrupted causing seizures. Highly connected nodes in these networks are epilepsy surgery targets. Here, we assess whether functional connectivity (FC) using intracranial electroencephalography can quantify brain regions epileptogenicity and predict surgical outcome in children with drug resistant epilepsy (DRE). We computed FC between electrodes on different states (i.e. interictal without spikes, interictal with spikes, pre-ictal, ictal, and post-ictal) and frequency bands. We then estimated the electrodes’ nodal strength. We compared nodal strength between states, inside and outside resection for good- (n = 22, Engel I) and poor-outcome (n = 9, Engel II–IV) patients, respectively, and tested their utility to predict the epileptogenic zone and outcome. We observed a hierarchical epileptogenic organization among states for nodal strength: lower FC during interictal and pre-ictal states followed by higher FC during ictal and post-ictal states (*p *< 0.05). We further observed higher FC inside resection (*p *< 0.05) for good-outcome patients on different states and bands, and no differences for poor-outcome patients. Resection of nodes with high FC was predictive of outcome (positive and negative predictive values: 47–100%). Our findings suggest that FC can discriminate epileptogenic states and predict outcome in patients with DRE.

## Introduction

The normal brain is increasingly seen as a dynamic system dependent on the integrity of structural and functional networks. Converging evidence from both animal and human studies has shown that a disruption of these networks may lead to epilepsy^1–4^. Even for focal epilepsy, seizure-generating tissues are not simple epileptogenic foci but are involved in microscale to macroscale mechanisms^5^. To accurately localize the epileptogenic focus, it is critical to identify functional abnormalities between regions, which are involved in the generation and propagation of epileptogenic activity^6–9^. Functional connectivity (FC), mostly estimated in intracranial electroencephalography (iEEG), has increasingly attracted the attention of researchers working on epilepsy. FC is defined as the temporal dependency of neuronal activation patterns of anatomically distant regions^10^ reflecting how different brain areas coordinate their activities^11^. Within this framework, iEEG contacts can be considered as *nodes*, while the connectivity between contacts as *edges*^8,12^. This relationship between *nodes* and *edges* are used to calculate the nodal strength defined for each *node* as the median of all *edges* connected to that *node*. Highly connected hubs (i.e. nodes with high nodal strength) are linked to epileptogenic regions since previous studies have shown increased FC within resection of good-outcome patients, and lower FC in distant non-epileptogenic areas^6,8,9,13,14^. Yet, there are still unanswered questions that are central to translating these iEEG-based FC tools to clinical practice^7,15,16^.

Current FC studies focus primarily on the analysis of resting-state iEEG recordings, which show synchronous neural activity in different regions in the absence of any task-related activity^17,18^. Most iEEG studies examined FC measures during either ictal or interictal states^7,19^. Yet, there are no studies so far assessing changes in FC across all epileptogenic states (e.g. interictal, pre-ictal, ictal, and post-ictal). Such FC alterations between epileptogenic states could provide critical insights into the pathophysiological mechanisms of epilepsy. Moreover, they might help in developing tools that delineate the epileptogenic zone (EZ) in patients with drug resistant epilepsy (DRE), monitor the effectiveness of antiseizure drugs, and predict seizures. There have been few attempts to develop an epileptogenic index (EI), which quantifies the “epileptogenic status” of brain regions based on both spectral and temporal properties of iEEG signals^13,15,20^. These studies mostly examined the transition from pre-ictal to ictal state and used tools that detect only fast oscillations. However, these tools are insufficient to detect slow patterns of seizure onset that account for 20–30% of iEEG patterns and are usually seen in patients with poor prognosis for surgical outcome^20^.

**Figure 1:**
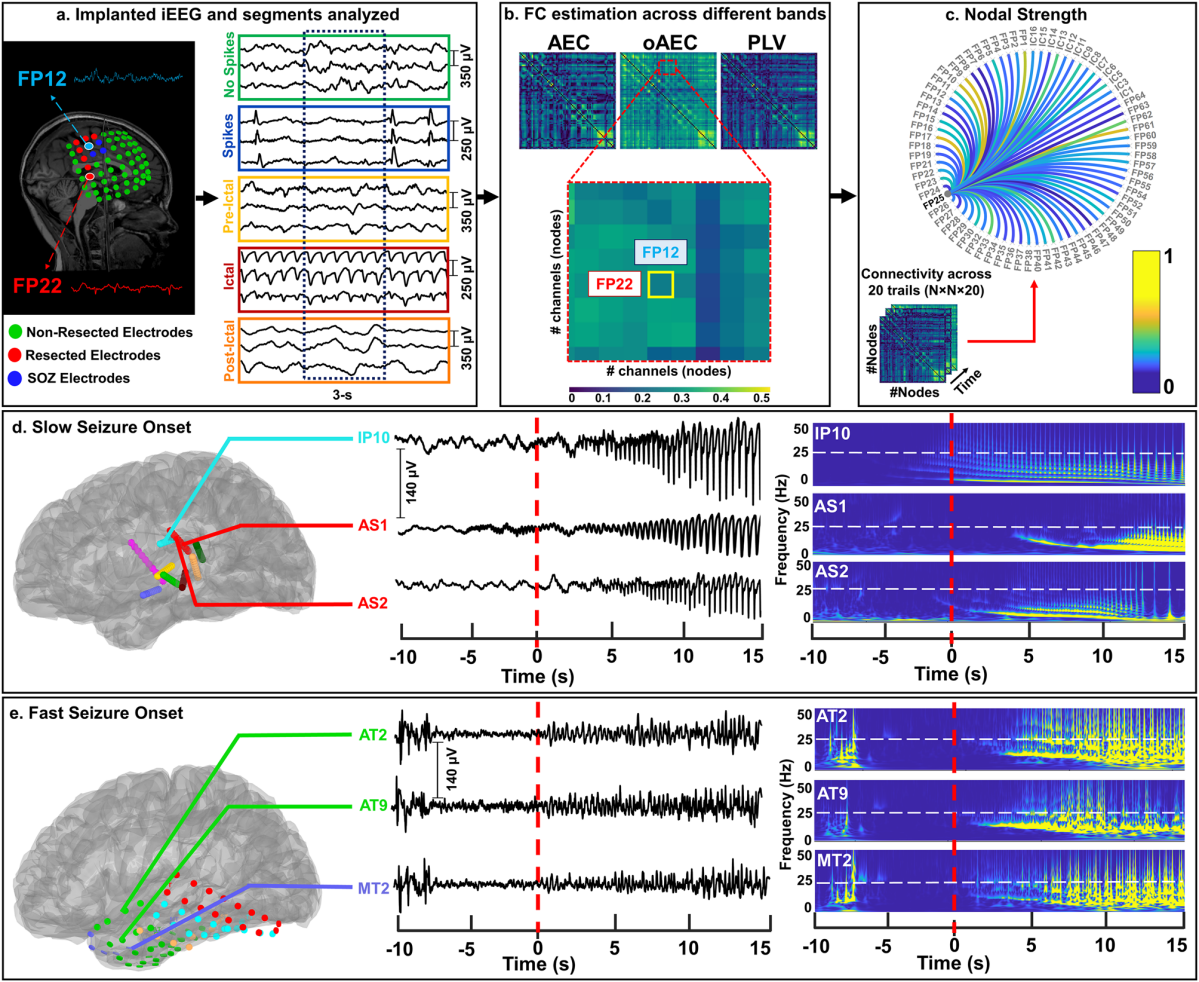
Functional connectivity (FC) measures extracted from intracranial EEG (iEEG) recordings. (**a**) *Left*: Placement of iEEG electrodes on a 2-year-old male (patient #4, Engel IA), which are defined as non-resected (displayed in green), resected (displayed in red), and identified as the seizure onset zone (displayed in blue); *Right*: Selection of 1-minute duration clips (20 non-overlapping segments of 3 s duration each) from the five epileptogenic states: interictal with no frank epileptiform activity (“No Spikes”, green-colored), interictal with frank epileptiform activity (“Spikes”, blue-colored), pre-ictal before the onset of a clinical seizure (“Pre-Ictal”, yellow-colored), ictal activity during a clinical seizure (“Ictal”, red-colored), and post-ictal activity after the end of a clinical seizure (“Post-Ictal”, orange-colored). (**b**) For each patient and epileptogenic state, Amplitude Envelope Correlation (AEC), orthogonalized Amplitude Envelope Correlation (oAEC), and Phase Locking Value (PLV) measures were computed for each segment and physiologically relevant frequency band. Rows and columns of each FC matrix represent the iEEG channels (or *nodes*) and each pixel represents the FC between pairs of channels; here, the FC between the FP12 and FP22 iEEG electrodes is highlighted. Each FC matrix is color-coded from low (displayed in blue) to high values (displayed in yellow). (**c**) For each channel, nodal strength was computed as the median of all the *edges* connected to the channel itself, across twenty segments. The FC graph is displayed as an example to highlight FC values between the FP25 iEEG channel with the others. (**d**) *Left*: Slow seizure onset (SSO) was recorded from the iEEG data of a 7-year-old female (patient #1, Engel IA); *Right*: Time-frequency analysis of iEEG time-series shows the theta-alpha sharp activity pattern characteristic of the SSO for this patient. (**e**) *Left*: Fast seizure onset (FSO) was recorded from the iEEG data of a 15-year-old male (patient #6, Engel IA); *Right*: Time-frequency analysis of iEEG time series shows the beta-gamma sharp activity pattern characteristic of the FSO for this patient.

In this study, we assess the ability of FC measures to quantify the “epileptogenic status” of a brain area at a specific time point and predict the outcome in children with DRE undergoing presurgical evaluation with iEEG. We hypothesize that FC measures on iEEG can discriminate between different epileptogenic states, identify hubs, and predict patients’ outcome. Particularly, we hypothesize that nodal strength follows a hierarchical epileptogenic organization across states: higher values correspond to a higher “epileptogenic status” of a brain area at a specific time point, whereas lower values to a lower one. Thus, nodal strength will have higher values during the ictal compared to pre-ictal and interictal states. Moreover, we hypothesize that these regions with increased nodal strength can identify the EZ, independently of the epileptogenic state, and their resection can predict outcome in patients with DRE. To test our hypotheses, we extracted iEEG epochs from interictal (with and without spikes), pre-ictal, ictal, and post-ictal states (Fig. 1a) and estimated undirected FC between regions using the Amplitude Envelope Correlation (AEC), orthogonalized version of AEC (oAEC), and Phase Locking Value (PLV) in physiologically relevant frequency bands (see experimental procedures) (Fig. 1b). For patients with stereotactic EEG (sEEG) implantation, we controlled for differences in sEEG spatial sampling (i.e. distance between electrodes) that may alter FC measures^6^. Then, we assessed the strength of each *node* (i.e. iEEG electrode) that reflects the FC between areas over time (Fig. 1c). Based on the appearance of rapid discharges before the seizure onset, we also differentiated seizures of each patient into two types, i.e. slow seizure onset (SSO) (Fig. 1d) and fast seizure onset (FSO) (Fig. 1e). Finally, we compared the nodal strength across states, inside and outside resection of good- and poor-outcome patients, respectively, examined the ability of FC in identifying epileptogenic *nodes* in good-outcome patients, and assessed the clinical utility of resecting highly connected hubs for predicting outcome.

## Results

We retrospectively analyzed iEEG data from 31 children (15 females, median age at surgery: 13 ± 5.81 years) with DRE. We dichotomized our patients based on their surgical outcome: good (n = 22, Engel I) and poor (n = 9, Engel II–IV). Patient demographics are summarized in Table 1. Five patients (16.13%) had normal MRI or abnormalities not related to epilepsy, and twenty-six patients (83.87%) had an anatomical lesion identified on MRI. The most common pathology observed among all patients was gliosis (n = 17, 54.84%), followed by malformation of cortical development (n = 7, 22.58%), hippocampal sclerosis (n = 2, 6.45%), polymicrogyria (n = 2, 6.45%), tumor (n = 1, 3.23%), and astrocytosis (n = 1, 3.23%); one patient had unknown pathology. All patients underwent iEEG implantation with either subdural [i.e., electrocorticography (ECoG); 12 patients], depth (i.e., sEEG; 7 patients), or both types (sEEG and ECoG; 12 patients). For patients with only depth or both types, we considered only the sEEG electrodes not included in the white matter. In total, we reviewed 891.45 minutes of iEEG data [median, interquartile range (IQR): 6.78 min, 5.41–13.75] from 3758 channels (median, IQR: 66 channels, 92–139) containing 586.43 and 305.01 minutes of interictal (with and without spikes) and ictal activity (pre-ictal, ictal, and post-ictal), respectively. Twenty-eight patients (90.32%) had iEEG implantation in the temporal lobe with 1008 out of 3758 iEEG electrodes (26.82%) predominantly located in this area. For each patient, we analyzed 1-min duration of recording from each epileptogenic state. Twenty patients (64.52%) had predominant SSO, whereas 11 patients (35.48%) had predominant FSO. We did not find differences between SSO and FSO patients in terms of age at epilepsy onset (*Wilcoxon rank-sum* test, *p *= 0.45), sex (*Fisher’s exact test*, *p *= 0.72), or outcome (*Fisher’s exact* test, *p *= 1.00). Seizures were recorded from the right hemisphere in five patients (16.13%), left hemisphere in 22 patients (70.97%), and both hemispheres in four patients (12.90%). All patients had clinical seizures analyzed with a focal origin, except two of them whose seizures underwent a bilateral spread (focal to bilateral tonic-clonic seizures). We did not find any correlation between the age at surgery and FC-based nodal strength (AEC, oAEC, and PLV) computed in different epileptogenic states (*p *> 0.05, *Spearman’s rank* correlation).

**Table 1:** Patient demographics.

#	Sex (F/M)	Age (years)	MRI findings	Pathology	Seizure type	Surgical procedure	SOZ contacts (#)	iEEG [#]	Laterality (L/R)	Engel (f/u, years)
1	F	7	FCD (T and Ins)	DEV	SSO	L-temporal resection	5	DE (90)	L	IB (8)
2	M	18	Tumor (T)	ACQ	SSO	L-frontotemporal resection	27	SE, DE (72+20)	L	IC (6)
3	F	16	FCD (T)	DEV	SSO	L- temporal resection	2	SE, DE (96+10)	L	IA (8)
4	M	2.2	Fr lesion	ACQ	FSO	L-frontotemporal resection	4	SE (80)	R	IA (6)
5	F	18	Normal	NL	SSO	Medial temporal lobectomy	12	SE (88)	L	IA (7)
6	M	15	Hippocampal Sclerosis (anterior T)	DEV	FSO	L-anterior medial temporal resection	2	SE (80)	L	IA (2)
7	M	5	FCD (T)	DEV	SSO	L- temporal resection	5	SE (96)	L	IA (1.5)
8	M	13	Encephalomalacia (P, superior T)	ACQ	SSO	Frontal parietal resection	25	SE, DE (72+30)	L	IC (2)
9	F	3	TSC (multifocal)	DEV	SSO	Inferior frontal resection	13	SE (120)	L/R	IA (2)
10	M	4	FCD (Fr)	DEV	FSO	Bilateral frontal resection	9	SE, DE (56+10)	L/R	IC (6)
11	M	22	FCD	DEV	SSO	L-frontotemporal resection	10	SE, DE (64+30)	L	IA (3)
12	M	13	FCD (Mesial T)	DEV	SSO	L-frontotemporal resection	9	SE (92)	L	IA (9)
13	M	10	PMG (Fr, P)	DEV	FSO	L-temporal resection	20	SE, DE (64+60)	L	IB (2)
14	M	4	R frontal pole, superior Fr gyrus	DEV	FSO	R-temporal resection	31	SE, DE (128+10)	L	IA (1.5)
15	F	8	FCD (Posterior Fr)	DEV	FSO	Left occipital ablation	15	DE (164)	L	IA (4)
16	F	18	FCD (Fr)	DEV	SSO	L-anterior frontal resection	9	SE, DE (72+40)	L	IA (2)
17	F	7	Parietal lesion	DEV	SSO	L-frontal resection	7	SE (100)	L	IA (1)
18	F	6	PMG (P, T, O)	DEV	SSO	R-occipital parietal resection	4	DE (196)	R	IA (1)
19	M	15	Normal	NL	SSO	L- occipital parietal ablation	48	DE (236)	L	IA (1)
20	M	13	Inferior Fr sulcus, pars Tr	DEV	FSO	L-frontal temporal resection	21	SE (96)	L	IA (1.5)
21	F	4	Fr Lesion	Unknown	FSO	R-parietal ablation	9	DE (162)	R	IA (1)
22	F	18	FCD (Fr)	MCD	SSO	L-frontal resection	6	SE, DE (144+10)	L/R	IB (1)
23	F	10	Hippocampal sclerosis	DEV	SSO	L-inferotemporal lobectomy	3	SE, DE (140)	L	IVB (6)
24	M	6	FCD (Fr)	DEV	FSO	L-frontal lobectomy	7	SE (120)	L	IIIA (1)
25	M	16	Normal	NL	FSO	A-temporal lobectomy	6	SE (88)	L/R	IIB (2)
26	M	16	Normal (mild gliosis)	NL	SSO	L-frontal resection	4	SE (88)	L	IIIA (5)
27	F	13	Normal	DEV	SSO	R-frontal resection	6	SE, DE (112+10)	L	III (2)
28	M	18	FCD	DEV	SSO	L-occipital ablation	20	DE (212)	L	IIA (6)
29	F	22	Trauma	ACQ	FSO	R-frontal resection	4	SE (120)	R	IIB (5)
30	F	7	FCD (Fr operculum)	DEV	SSO	R-temporal resection	5	SE, DE (72+40)	R	IIA(2.5)
31	F	15	Hippocampal Formation	ACQ	SSO	L-frontal ablation	33	DE (166)	L	IIIA (2)

ACQ acquired (i.e., stroke, neoplasm and traumatic brain injury); Age age at epilepsy surgery; DE depth electrodes (stereo EEG); DEV malformation of cortical development (i.e., focal cortical dysplasia (FCD), polymicrogyria, tuberous sclerosis complex, dysembryoplastic neuroepithelial tumor, and glioma); *F* female; *f/u* follow-up; *Fr* frontal; *FSO* fast seizure onset; *I* inferior; *L* left; *M* male; *NL* non-lesional; *O* occipital; *P* parietal; *PMG* polymicrogyria; *R* right; *SE* subdural electrodes (electrocorticography); *SSO* slow seizure onset; *T* temporal; *Tr* triangularis; *TSC* tuberous sclerosis complex.

### FC discriminates between interictal, pre-ictal, ictal, and post-ictal states

**Figure 2:**
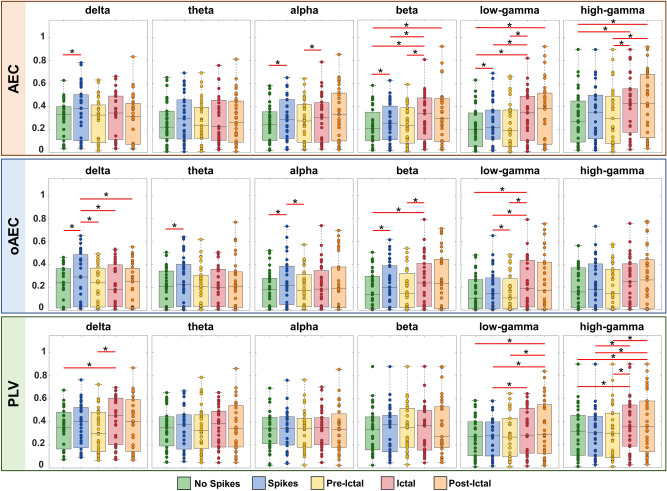
Nodal strength for different epileptogenic states in physiologically relevant frequency bands. Amplitude Envelope Correlation (*Top*), orthogonalized Amplitude Envelope Correlation (*middle*), and Phase Locking Value (PLV) (*bottom*) nodal strength computed for delta, theta, alpha, beta, low- and high-gamma bands on different epileptogenic states. Each epileptogenic state is color-coded: interictal activity with no spikes (“No Spikes”, green-colored), interictal activity with spikes (“Spikes”, blue-colored), pre-ictal activity before the onset of a clinical seizure (“Pre-Ictal”, yellow-colored), ictal activity during a clinical seizure (“Ictal”, red-colored), post-ictal activity after the end of a clinical seizure (“Post-Ictal”, orange-colored). Significant differences are marked with an asterisk (*) (*p *< 0.05, *Wilcoxon’s signed-rank* test). *P*-values are corrected for multiple comparisons using the false discovery rate method. In the box-plot diagrams, the horizontal line indicates the median value, lower and upper edges represent the 25th and 75th percentiles, whiskers extend to the minimum and maximum values (excluding outliers) and points outside the whiskers represent the outliers (i.e., values that are at least 1.5 times the interquartile range below the 25th percentile or above the 75th percentile).

For all patients, we generated a patient-specific FC-based network using nodal strength. From each FC (i.e., AEC, oAEC, and PLV) matrix, we computed the nodal strength for each iEEG electrode and averaged these values to obtain a unique median nodal strength value for each patient, frequency band, and epileptogenic state. Finally, we compared these median nodal strength values across states for different bands and assessed the magnitude of their difference. A similar comparison across states was also performed considering iEEG electrodes located in the intra- and extra-temporal areas. For AEC, we observed higher FC of interictal data with spikes compared to interictal data with no spikes (*p *< 0.05) for delta (14%), alpha (8%), beta (10%), and low-gamma (6%) bands (Fig. 2). We also observed increased AEC nodal strength of ictal state compared to pre-ictal (*p *< 0.05) for alpha (9%), beta (10%), low-gamma (30%), and high-gamma (4%) bands. High AEC nodal strength was observed for ictal compared to interictal state with no spikes [beta (13%), low-gamma and high-gamma bands (17% and 18%, respectively)] and with spikes [beta (5%) and low-gamma (9%) bands] (*p *< 0.05). AEC nodal strength was higher in post-ictal compared to interictal state with no spikes [beta (14%), low-gamma (16%), and high-gamma (18%) bands] and to pre-ictal state [high-gamma band (9%)] (*p *< 0.05). For oAEC, we observed increased FC of interictal with spikes compared to interictal with no spikes [delta (20%), theta (19%), alpha (13%), and beta (18%) bands], pre-ictal [delta (13%), alpha (15%), and low-gamma (11%) bands], as well as to ictal (15%) and post-ictal (13%) states (delta band) (*p *< 0.05) (Fig. 2). oAEC nodal strength of the ictal state had higher values than interictal with no spikes and pre-ictal states for beta (15% and 25%, respectively) and low-gamma (20% and 29%, respectively) bands, as well as compared to interictal state with spikes low-gamma band (18%) (*p *< 0.05) (Fig. 2). For PLV, nodal strength of ictal state was higher (*p *< 0.05) compared to interictal with no spikes and pre-ictal states [for delta (7% and 10%, respectively) and high-gamma (20% and 4%, respectively) bands] and to interictal state with spikes [low-gamma (6%) and high-gamma (7%) bands] (Fig. 2). Moreover, we observed increased PLV nodal strength of post-ictal state compared to interictal with no spikes, interictal with spikes, and pre-ictal states for low-gamma (10%, 12%, and 8%, respectively) and high-gamma (13%, 10%, and 7%, respectively) bands (*p *< 0.05) (Fig. 2). *P*-values were corrected for multiple comparisons using the False Discovery Rate (FDR) method. Further, we compared AEC, oAEC, and PLV nodal strength values separately for intra- and extra-temporal electrodes across all states and bands, as well as between these two types (*Wilcoxon signed-rank* test). For each electrode’s type, we observed differences among states and bands (*p *< 0.05, FDR corrected) (Supplementary Figs. S1 and S2), but no differences when comparing nodal strength between these two types.

To assess whether the results are affected by bias introduced by the type of the implantation performed, we compared median nodal strength across states and bands for patients having only sEEG, ECoG, and both types, respectively. We found differences (*p *< 0.05) of nodal strength for patients with only ECoG and both types (Supplementary Table S1), but no difference for patients with only sEEG. Further, significant differences (*p *< 0.05) were observed when comparing median nodal strength between patients with only sEEG or ECoG (Supplementary Table S2). No differences in nodal strength were found between ECoG and sEEG in patients who had both types. To examine to what degree the propagation of seizures may affect our findings, we compared the averaged ictal nodal strength between electrodes involved and not involved in seizure evolution (see experimental procedures) (*Wilcoxon signed-rank* test) and observed no differences (Supplementary Fig. S3).

### FC delineates epileptogenic nodes of the network

**Figure 3:**
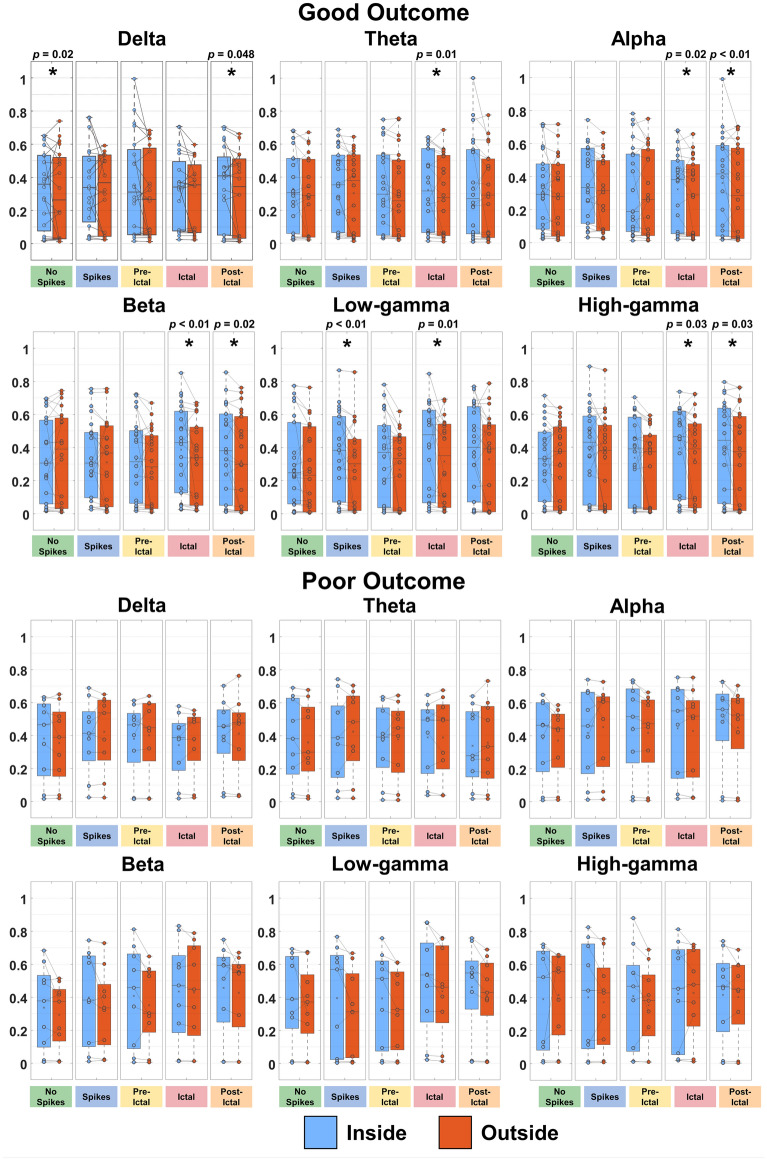
Orthogonalized amplitude envelope correlation (oAEC) nodal strength inside and outside resection for good- and poor-outcome patients in physiologically relevant frequency bands. oAEC nodal strength values computed from electrodes located inside vs. outside resection for all epileptogenic states in physiologically relevant frequency bands for good (n=22, Engel I) and poor (n=9, Engel II–IV) surgical outcomes, separately. Nodal strength values inside the resection are blue-colored; nodal strength values outside the resection are orange-colored. Significant differences are marked with an asterisk (*) (*p *< 0.05, *Wilcoxon signed-rank* test). In the box-plot diagrams, the horizontal line indicates the median value, the cross (×) sign indicates the mean, the lower and upper edges represent the 25th and 75th percentiles, and the whiskers extend to the minimum and maximum values.

**Figure 4:**
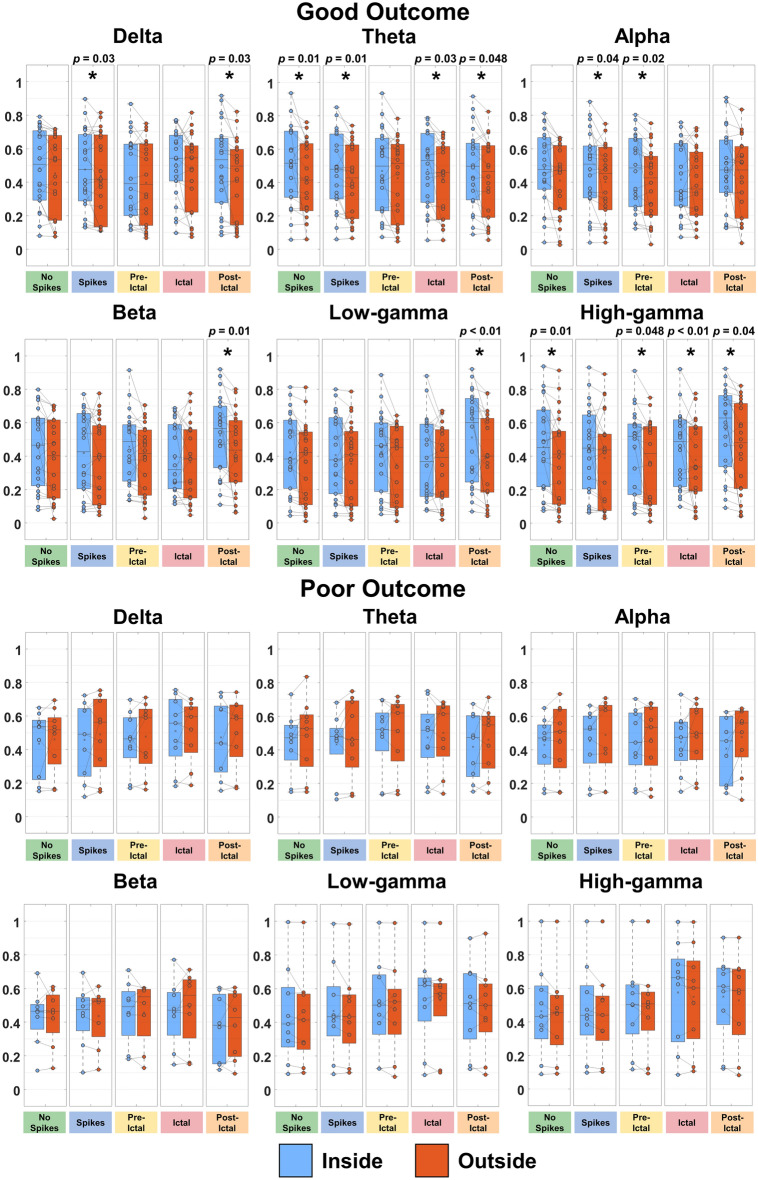
Phase locking values (PLV) nodal strength inside and outside resection for good- and poor-outcome patients in physiologically relevant frequency bands. PLV nodal strength values computed from electrodes located inside vs. outside resection for all epileptogenic states in physiologically relevant frequency bands for good (n=22, Engel I) and poor (n=9, Engel II–IV) surgical outcomes, separately. Nodal strength values inside the resection are blue-colored; nodal strength values outside the resection are orange-colored. Significant differences are marked with an asterisk (*) (*p *< 0.05, *Wilcoxon* *signed-rank* test). In the box-plot diagrams, the horizontal line indicates the median value, the cross (×) sign indicates the mean, the lower and upper edges represent the 25th and 75th percentiles, and the whiskers extend to the minimum and maximum values.

To quantify the relationships between FC and the EZ, we compared nodal strength of electrodes inside vs. outside resection, as well as inside vs. outside the clinically defined seizure onset zone (SOZ), for good- and poor-outcome patients, separately. For AEC, we observed higher nodal strength inside (vs. outside) resection of good-outcome patients (*p *< 0.05) for the states: (i) interictal with no spikes for alpha and beta bands; (ii) interictal with spikes for delta, alpha, beta, and high-gamma bands; (iii) pre-ictal for theta, low-gamma, and high-gamma bands; (iv) ictal for theta band; and (v) post-ictal for alpha, beta, low-gamma, and high-gamma bands (Supplementary Fig. S4). Moreover, AEC nodal strength of post-ictal state was higher inside (vs. outside) SOZ for alpha and high-gamma bands of good-outcome patients (*p *< 0.05) (Supplementary Fig. S5). For oAEC, we observed increased nodal strength inside (vs. outside) resection of good-outcome patients (*p *< 0.05) for the states: (i) interictal with no spikes for delta band; (ii) interictal with spikes for low-gamma band; (iii) ictal for theta, alpha, beta, low-gamma, and high-gamma bands; and (iv) post-ictal for delta, alpha, beta, and high-gamma bands (Fig. 3). We also found high nodal strength inside (vs. outside) the SOZ of good-outcome patients (*p *< 0.05) for the states: (i) interictal with spikes for alpha, low-gamma, and high-gamma bands; (ii) pre-ictal for theta and beta bands; and (iii) post-ictal for alpha, beta, low-gamma, and high-gamma bands (Supplementary Fig. S6). For PLV, we observed higher nodal strength inside resection (vs. outside) of good-outcome patients (*p *< 0.05) for the states: (i) interictal with no spikes for theta and high-gamma bands; (ii) interictal with spikes for delta, theta, and alpha bands; (iii) pre-ictal for alpha and high-gamma bands; (iv) ictal for the theta and high-gamma bands; and (v) post-ictal for delta, theta, beta, low-gamma, and high-gamma bands (Fig. 4). Moreover, we observed increased PLV nodal strength inside SOZ (vs. outside) of good-outcome patients (*p *< 0.05) for the states: (i) interictal with spikes and pre-ictal for delta band; and (ii) post-ictal for theta, beta, and low-gamma bands (Supplementary Fig. S7). We did not find differences between inside vs. outside the resection and SOZ for poor-outcome patients (Figs. 3, 4 and Supplementary Figs. S4–7).

**Figure 5:**
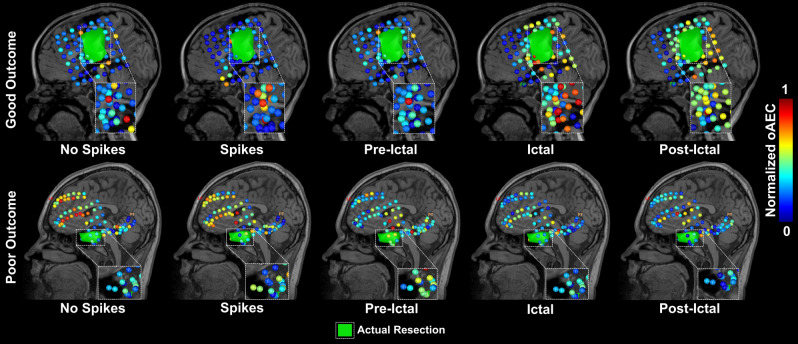
Orthogonalized amplitude envelope correlation (oAEC) nodal strength across different epileptogenic states at the patient level. Using the magnetic resonance imaging scans of a 5-year-old seizure-free male (patient #7, Engel IA) (*top*) and a 16-year-old not seizure-free male (patient #26, Engel IIIA) (*bottom*), we displayed the oAEC nodal strength computed for all epileptogenic states (for the delta band) from interictal state with no spikes (“No Spikes”, *left*) to the post-ictal state (“Post-Ictal”, *right*). The oAEC nodal strength values are color-coded: high oAEC nodal strength values are displayed in red; low oAEC nodal strength values are displayed in blue. Resection zone is displayed as a green volume. “Spikes”: interictal activity with spikes; “Pre-Ictal”: pre-ictal activity before the onset of a clinical seizure; “Ictal”: ictal activity during a clinical seizure; “Post-Ictal”: post-ictal activity after the end of a clinical seizure.

To highlight the relationship of increased nodal strength with the EZ, we represented oAEC nodal strength (delta band) for a patient with good (patient #7, Engel IA) and poor outcome (#26, Engel IIIA), respectively (Fig. 5), across all states. For the good-outcome patient, increased FC was observed within the margins of resected-areas (i.e., EZ) and lower FC in distant brain regions. Contrarily for the poor-outcome patient, increased FC values were scattered in different resected and non-resected brain regions.

### Increased FC inside epileptogenic regions

To determine whether there is an increase in FC within epileptogenic regions, we conducted two additional analyses at the electrode level. For each patient and type of analysis (between vs. within connectivity), we computed the average nodal strength for the epileptogenic (inside resection) and non-epileptogenic (outside resection) regions, respectively, and compared these values for each state and band. These analyses were specifically performed on patients with good outcomes who underwent sEEG implantation. We included sEEG electrodes from patients with only sEEG implantation, as well as those who had both ECoG and sEEG implantations (see experimental procedure). Across all patients, we noted that epileptogenic regions often had higher oAEC nodal strength with other regions (“between connectivity”) for delta, theta, low-gamma, and high-gamma bands (Supplementary Fig. S8). Particularly, between connectivity (i.e., oAEC nodal strength) was higher in epileptogenic compared to non-epileptogenic regions for the states: (i) interictal with no spikes for theta, low-gamma, and high-gamma bands (*p *= 0.013, *p *= 0.005, and *p *= 0.037, respectively); (ii) interictal with spikes for low-gamma band (*p *= 0.031); (iii) pre-ictal for delta band (*p *= 0.027); and (iv) ictal for low-gamma (*p *= 0.048) (Supplementary Fig. S8). We did not observe FC differences between epileptogenic and non-epileptogenic regions within the single brain regions (“within connectivity”) across all patients.

### Identification of epileptogenic nodes

**Figure 6:**
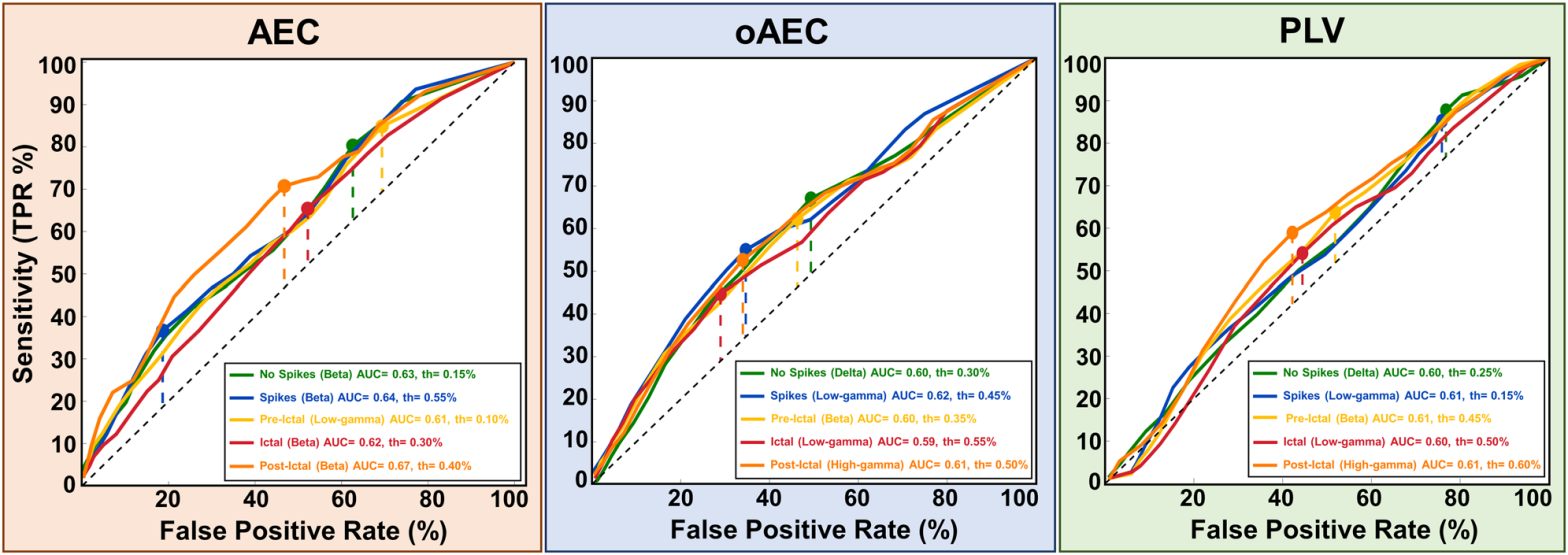
Classification between epileptogenic and non-epileptogenic nodes in good-outcome patients. For each frequency band, the receiver operating characteristic (ROC) curves estimated from the amplitude envelope correlation (AEC), orthogonalized AEC (oAEC), and phase locking value (PLV)-based nodal strength computed for each node in patients with good surgical outcome (n=22, Engel I) in different epileptogenic states. The highest areas under the ROC curve (AUC) for each epileptogenic state obtained only in specific frequency bands are color-coded: interictal activity with no spikes (“No Spikes”, green-colored), interictal activity with spikes (“Spikes”, blue-colored), pre-ictal activity before the onset of a clinical seizure (“Pre-Ictal”, yellow-colored), ictal activity during a clinical seizure (“Ictal, red-colored), post-ictal activity after the end of a clinical seizure (“Post-Ictal”, orange-colored). The black-colored dash diagonal line represents the performance of FC measures in discriminating the epileptogenicity of nodes that is no better than a random chance. The colored dash diagonal lines represent the maximum Youden index (J) for each epileptogenic state, respectively. The colored-points on the ROC curves represent the cut-off thresholds for each epileptogenic state, respectively. FPR= false positive rate (i.e., 1- specificity); *TPR* true positive rate (i.e., sensitivity); *th* threshold.

To assess whether resection of highly connected hubs (i.e. nodes with high nodal strength) can delineate the EZ, we examined whether these nodes were located inside (or outside) the EZ in good-outcome patients. Particularly, we performed a receiver operating characteristic (ROC) curve analysis to assess the performance of AEC, oAEC, and PLV nodal strength in distinguishing between epileptogenic (inside resection) and non-epileptogenic (outside resection) electrodes of good-outcome patients. We found that AEC identified epileptogenic nodes in good-outcome patients [area under the curve (AUC) ≥ 0.61] for the following states: (i) interictal with no spikes for beta band; (ii) interictal with spikes for beta band; (iii) pre-ictal for low-gamma band; (iv) ictal for beta band; and (v) post-ictal for beta band (Fig. 6). Moreover, we observed that oAEC identified epileptogenic nodes in good-outcome patients (AUC ≥ 0.59) for the following states: (i) interictal with no spikes for delta band; (ii) interictal with spikes for low-gamma band; (iii) pre-ictal for beta band; (iv) ictal for low-gamma band; and (v) post-ictal for high-gamma band (Fig. 6). Finally, we found that PLV identified epileptogenic nodes in good-outcome patients (AUC ≥ 0.60) for the following states: (i) interictal with no spikes for delta band; (ii) interictal with spikes for low-gamma band; (iii) pre-ictal for beta band; (iv) ictal for low-gamma band; and (v) post-ictal for high-gamma band (Fig. 6).

### Prediction of surgical outcome

**Figure 7:**
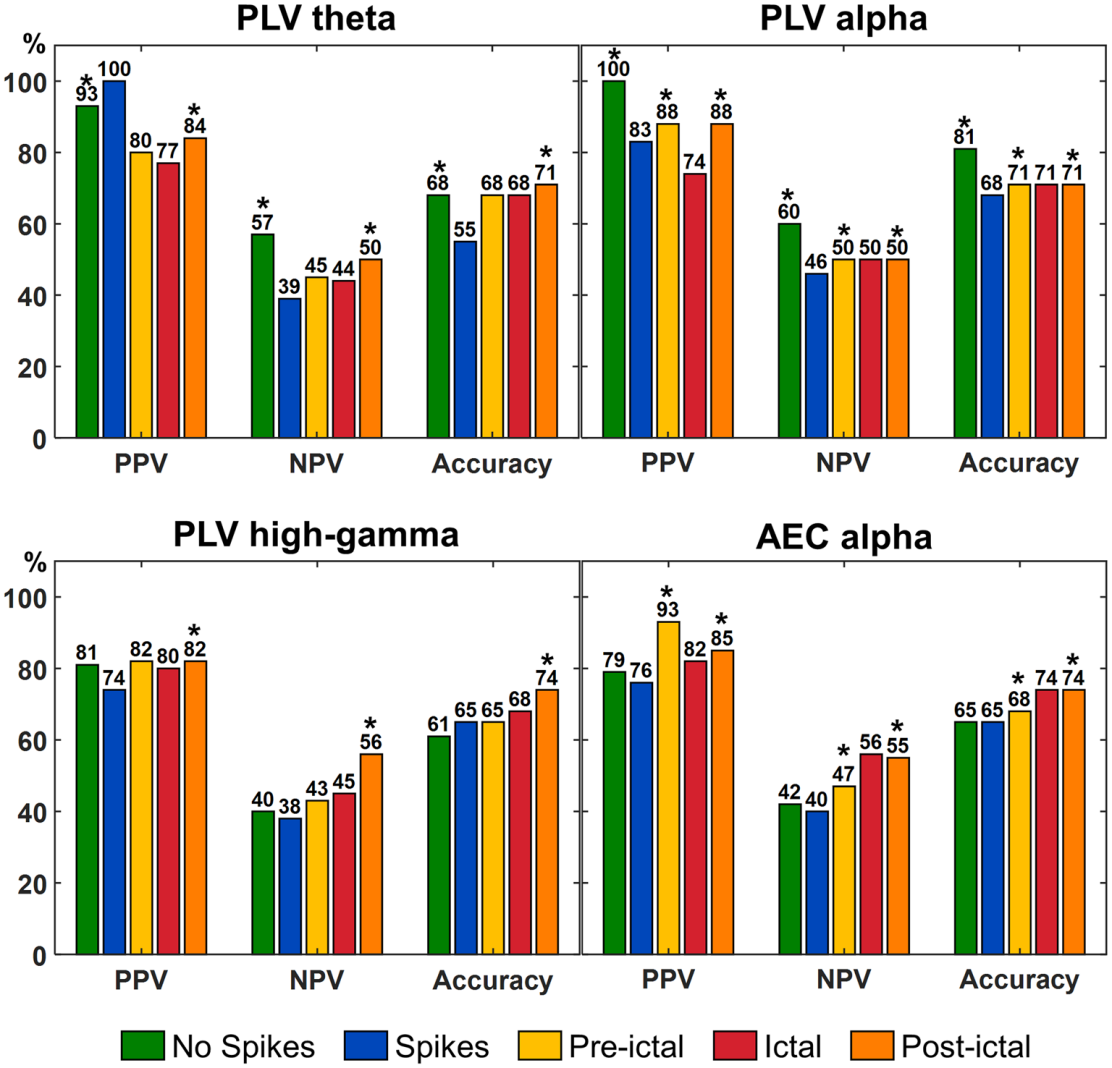
Surgical outcome prediction results for different epileptogenic states. Resection of highly connected hubs (i.e., nodes with high nodal strength) computed from phase locking value (PLV) (for the theta, alpha, and high-gamma bands) and amplitude envelope correlation (AEC) (for the alpha band) was associated with good surgical outcome for different epileptogenic states. Each epileptogenic state is color-coded: interictal activity with no spikes (“No Spikes”, green-colored), interictal activity with spikes (“Spikes”, blue-colored), pre-ictal activity before the onset of a clinical seizure (“Pre-Ictal”, yellow-colored), ictal activity during a clinical seizure (“Ictal, red-colored), post-ictal activity after the end of a clinical seizure (“Post-Ictal”, orange-colored). Significant differences are marked with an asterisk (*) (*p *< 0.05, *Fisher’s exact* test). *PPV* positive predictive value, *NPV* negative predictive value.

To assess the prognostic values of resecting highly connected hubs (i.e., nodes with nodal strength above threshold), we defined whether their overlap with resection predicted good-outcome or not (*Fisher’s exact* test). The optimal connectivity thresholds for each FC metric (i.e., AEC, oAEC, and PLV) and their relative percentages of overlap with resection were identified as the ones that provided the maximum Youden index (J) obtained from the ROC curves. PLV nodal strength was predictive of outcome for the states: (i) interictal with no spikes for theta [*p *= 0.02; optimal FC threshold (thr) = 0.6; overlap = 35%] and alpha (*p *= 0.0002; thr = 0.65; overlap = 15%) bands; (ii) pre-ictal for alpha band (*p *= 0.04; thr = 0.7; overlap = 10%); and (iii) post-ictal for theta (*p *= 0.02; thr = 0.7; overlap = 5%), alpha (*p *= 0.04; thr = 0.7; overlap = 5%), and high-gamma (*p*=0.003; thr = 0.75; overlap = 5%) bands (Fig. 7). Similarly, AEC predicted outcome for the pre-ictal (*p *= 0.02; thr = 0.8; overlap = 5%) and post-ictal (*p *= 0.02; thr = 0.7; overlap = 5%) states for alpha band (Fig. 7).

Based on the seizure classification of each patient, we further assessed the prognostic value of resecting hubs separately for patients with SSO and FSO on ictal state (*Fisher’s exact* test). For patients with FSO, we observed that oAEC (low-gamma band) [positive predictive value (PPV) = 75%; negative predictive value (NPV) = 66%; accuracy = 72%; *p *= 0.04; thr = 0.7; overlap = 5%] and PLV (for alpha band) (PPV = 66%; NPV = 50%; accuracy = 63%; *p *= 0.01; thr = 0.35; overlap = 5%) were predictive of outcome. For patients with SSO, we observed that AEC (theta band) (PPV = 82%; NPV = 66%; accuracy = 72%; *p *= 0.04; thr = 0.7; overlap = 5%) and PLV (alpha band) (PPV = 100%; NPV = 31%; accuracy = 45%; *p *= 0.03; thr = 0.65; overlap = 45%) were predictive of outcome.

To assess whether hubs had higher overlap with resection in good- compared to poor-outcome patients, we considered only those electrodes with nodal strength higher than their respective connectivity thresholds for AEC, oAEC, and PLV, respectively. Particularly, we computed the percentages of overlap of these hubs with resection of good- and poor-outcome patients, respectively, and compared these values using *Wilcoxon rank-sum* test. Moreover, we applied the same procedure to the SOZ, considering its overlap with those hubs for good- and poor-outcome patients, respectively. We observed an increased overlap of hubs with resection in good- compared to poor-outcome patients for the: (i) AEC nodal strength on interictal state with no spikes (beta band) (good-outcome: 67%; poor-outcome: 8%; *p *= 0.005); and (ii) PLV nodal strength on ictal state (delta band) (good-outcome: 67%; poor-outcome: 15%; *p *= 0.002). Moreover, we observed an increased overlap of hubs with the SOZ in good- compared to poor-outcome patients for the oAEC (low-gamma band) (good-outcome: 73%; poor-outcome: 9%, *p *= 0.0002) and PLV (delta band) (good-outcome: 76%; poor-outcome: 19%; *p *= 0.002) on interictal state with spikes, respectively.

## Discussion

In this study, we demonstrate that iEEG-based FC measures can discriminate epileptogenic states, identify highly connected hubs, and predict surgical outcome in children with DRE. Our notion derived from our main findings: (i) AEC, oAEC, and PLV nodal strength followed a hierarchical organization among states: lower FC during interictal and pre-ictal states and higher FC during ictal and post-ictal states (most prominent findings in low-gamma, high-gamma, and beta bands); (ii) increased nodal strength during ictal compared to pre-ictal state for several bands (AEC: alpha, beta, low-gamma and high-gamma; oAEC: beta and low-gamma; PLV: delta and high-gamma); (iii) increased nodal strength during interictal with spikes compared to no spikes for several bands (AEC: delta, alpha, beta, and low-gamma; oAEC: delta, theta, alpha, and beta); (iv) higher AEC, oAEC, and PLV nodal strength for different states recorded from electrodes inside resection for patients with good outcome (most prominent findings in theta, alpha, beta, and high-gamma bands); (v) increased oAEC *between connectivity* in the epileptogenic regions of good-outcome patients, calculated from sEEG electrodes, during interictal (with and without spikes), pre-ictal, and ictal states; (vi) resection of highly connected hubs was associated with good outcome for interictal, pre-ictal, and post-ictal states in several bands (AEC: alpha; PLV: theta, alpha, and high-gamma); (vii) resection of oAEC and PLV ictal nodal strength was associated with good outcome for patients with FSO; and (viii) resection of AEC and PLV ictal nodal strength was associated with good outcome for patients with SSO.

Interictal spikes and seizures are not always localized to a specific brain region but are often recorded at multiple regions, suggesting that the epileptogenic activity rapidly propagates to distant regions of the network^21–23^. Appropriate tools, such as FC measures, are therefore necessary to quantify the underlying proprieties of these networks. So far, most of the iEEG-based FC studies lack of a comparative analysis of the pre-ictal and ictal states with other states, such as interictal and post-ictal^13,20,24^. Here, we fill this gap by extensively exploring FC patterns across five epileptogenic states using three undirected FC measures (AEC, oAEC, and PLV). We observed increased nodal strength during interictal with spikes compared to no spikes for several bands (Fig. 2). Based on our findings, we provided evidence that during interictal state with spikes there are mechanisms triggering an increased interaction (i.e. FC) between regions. These results are in line with previous studies showing increased FC particularly when prominent interictal events occur or spread to other brain region^25–29^.

Scalp EEG and iEEG studies have shown that during the pre-ictal state gradual changes of electrophysiological patterns occur before the seizure manifestation^30^. During the transition from interictal to pre-ictal states, abnormal neural synchronizations lead regions to approach a similar dynamical state^31^. These alterations seen in the pre-ictal state might explain the mechanism leading to a seizure and potentially predict its occurrence^32^. Increased connectivity estimated through phase synchronization^33^ and PLV^34^ was observed during the interictal state and low values during pre-ictal state. Similarly, we found increased oAEC nodal strength during the transition from the interictal with spikes to pre-ictal state.

Strong excitatory input to pyramidal cells disrupts the excitatory-inhibitory balance during seizure onset, resulting in hypersynchronous abnormal discharges among neurons that disrupt brain functions^30,35,36^. Previous EEG studies have shown that the synchronicity of neural activity is enhanced during ictal compared to pre-ictal states^37–39^. Since FC quantifies the synchronicity among regions, we expect FC to be lower before seizure onset and higher later during the seizure course. Our findings support this notion since AEC, oAEC, and PLV nodal strength was increased during ictal compared to pre-ictal state.

It is logical to expect a decrease in neuronal synchronization after seizure termination. Here, we found FC decrease during post-ictal state compared to interictal spikes for lower bands (delta) (Fig. 2). The mechanism driving the post-ictal state is poorly understood but the leading theory is that subcortical nuclei are dormant when the seizure ends, and deep brain structures connected to cortical seizure foci are disrupted in the ictal phase, leading to a window of subcortical deactivation and decreased FC in post-ictal state^40^. Higher bands (low-gamma and high-gamma) showed increased post-ictal FC compared to interictal state with spikes (Fig. 2). This may represent a rebound response following the suppression of these frequencies during the seizure^41^. Animal studies have suggested that these changes may also reflect an attempt by the brain to restore normal activity following the disruption caused by the seizure^42^. Additionally, we found differences in nodal strength across epileptogenic states between patients with sEEG and ECoG implantation (see Supplementary Table S2). Contrary, no such differences were observed for sEEG and ECoG in patients with both implantations. The observed differences between different implantation modalities can be attributed to the heterogeneity of sEEG implantation, which can manifest in the following ways: (i) differences in the electrode implantation in terms of number; (ii) different assortments of anatomical targets and electrode spacing; (iii) differences in spatial orientation and density of electrode implantations; and (iv) differences in the level of bilateral implantation between the two modalities^43^. Future studies comparing the two modalities should include an equal number of sEEG and ECoG electrodes in the same anatomical locations to reduce biases^43^.

Previous iEEG studies have shown that FC coupled with graph-theory principles is a potential biomarker for delineating the EZ^8,9,12,13,18,19,44^. Yet only few studies have explored the clinical utility of FC to predict outcome^6,7,9,19,45^. Current clinical practice dictates the EZ delineation through its approximation with the SOZ defined by iEEG monitoring^46^. However, the SOZ may not represent the full EZ extent, and its delineation requires the recording of several stereotyped seizures at the expense of human and financial resources^47–49^. Here, we used FC to delineate the EZ independently from the epileptogenic state. We assumed that in good-outcome patients, vital parts of the EZ were contained within resection; contrarily, in poor-outcome patients, the resection did not include critical hubs. Prior FC studies explored the clinical utility of nodal strength for identifying the EZ: ‘hyper-connectivity’ was observed within epileptogenic foci and ‘hypo-connectivity’ outside these foci^5,8,19^. Similarly, we observed higher AEC and PLV nodal strength within resection (vs. outside) in almost all states for good-outcome patients, and no differences for poor-outcome patients. These findings hold true for both interictal states (with and without spikes) across different bands (Supplementary Fig. S4, Figs. 3 and 4). ROC analysis showed that FC can identify hubs for both interictal states (AUC range: 0.60–0.64) (Fig. 6). Moreover, FC can predict outcome with PPV ≥ 93% and NPV ≥ 57% on interictal data without spikes (Fig. 7). Taken together, FC can delineate the EZ and predict outcome without waiting for a seizure to occur or even in the absence of epileptiform activity in iEEG.

Our study has some limitations. We computed FC on iEEG data, which have limited spatial sampling due to iEEG placement^8^. Future studies may overcome these limitations using either electric source imaging or full-head noninvasive methods^50,51^. Since iEEG studies lack healthy control data, differences in physiological FC in healthy vs. epileptic brains are still unexplored. We visually examined spikes to prevent misclassifying artifacts as epileptic oscillations. However, we did not evaluate the signal energy in different bands during either interictal or ictal periods. Future research measuring the energy accumulation along with altered FC during epileptogenic states could provide insights into epileptic synchronization mechanisms. Additionally, instances of “frequency-locking” needs to be identified on iEEG data, which can be misinterpreted as increases in the FC at certain frequency sub-bands^52^. We further explored FC only on common average referenced data; future investigations should address the impact that other referencing techniques may have on FC. Moreover, our findings do not provide information regarding the directionality of information flow between epileptogenic and non-epileptogenic regions. Directed connectivity measures may help to map propagation phenomena of ictal and interictal activity^53,54^. We also used only one simple measure of graph-theory (i.e., nodal strength) extensively studied in epilepsy^8,13,55^ that might not fully interpret the complexity of abnormal brain networks^56^. Our findings suggest that AEC-based nodal strength is more effective than oAEC and PLV in identifying epileptogenic nodes in most epileptogenic states, with moderate AUC values ranging from 0.61 to 0.67. Previous studies have compared various FC measures and evaluated their reliability and accuracy in different signal generation models^52^. Regression methods have demonstrated good or average performance and sensitivity to the coupling parameter in all test models. In contrast, phase synchrony methods were found to be insensitive to an increase in the coupling parameter^52^. To determine the most suitable FC measure for clinical settings, future studies should integrate ensemble learning algorithms^57^. This approach will reduce dependence on results obtained from a single method and parameters, thereby increasing confidence in the final connectivity estimates. By utilizing these techniques, the overall reliability and accuracy of FC measures can be improved, ultimately enhancing their utility in clinical applications. We also found that AEC and PLV (for alpha band) predicted outcome with a low NPV (range: 47–50). However, in most poor-outcome patients of our cohort, the attempted resection was “incomplete” given either the overlap or proximity of the epileptogenic tissue to the eloquent cortex (e.g., language Broca’s area, supplementary and primary motor areas). Thus, the clinically defined EZ may have not been completely resected (or laser ablated) without permanently compromising their neurological functions. Finally, our patients were maintained on different doses of several antiseizure drugs tapered by our epilepsy surgery team during iEEG monitoring. Since drug load scores were not available in our cohort, we did not control for the effects that antiseizure drugs^45^ may have on our results.

In conclusion, we reveal for the first time that FC measures can discriminate epileptogenic states with increased values mainly observed when prominent electrophysiological events occur, such as interictal (i.e., spikes) and ictal epileptiform discharges. We provide a promising epilepsy biomarker for the EZ delineation and prediction of surgical outcome for patients with DRE. Such a biomarker may augment surgical planning and help develop neurostimulation devices that can prevent clinical epileptic attacks.

## Methods

### Study setting and participants

We retrospectively reviewed children with DRE who underwent resective neurosurgery at Boston Children’s Hospital (BCH) after long-term iEEG monitoring between June 2011 and June 2018. The cohort was selected based on: (i) at least one seizure in iEEG; (ii) availability of at least 1-min iEEG segments of interictal activity without spikes, interictal activity with spikes, as well as pre-ictal, ictal, and post-ictal states; (iii) availability of post-implantation computerized tomography (CT) and pre-operative MRI; (iv) accurate information about the resection volume and the clinically defined SOZ; and (v) availability of post-surgical outcomes ≥ 1 year. The procedures for the study were sanctioned by the North Texas Regional IRB (2019-166; PI: C. Papadelis), that waived the need for informed consent considering the study’s retrospective nature. All methods and analyses were performed in accordance with relevant guidelines and regulations.

### Long-term iEEG acquisition

Invasive monitoring using subdural and depth electrodes, or both types were performed with the XLTEK NeuroWorks (Natus Inc., USA) recording system for several days. Subdural grid and strip electrodes (Ad-Tech., USA) had a diameter of 2.3 mm with a distance between them of 10 mm, while depth electrodes had 10 linearly arranged contacts with a diameter of 1.1 mm and an inter-distance of 3–5 mm. The number, location, and type of implantation were prospectively decided by clinicians independently from this study. For each patient, we obtained artifact-free portions of iEEG data (sampling rate ≥ 500 Hz).

### Co-registration of iEEG electrodes

Using *Brainstorm*^58^, we coregistered the post-implantation CT (voxel size=0.5 ×0.5×0.5 mm^3^) and pre-operative MRI. Pre-operative anatomical MRI was acquired with magnetization-prepared rapid acquisition gradient-echo sequences using high resolution 3T scanner (TIM TRIO, Siemens AG). Each electrode’s location was confirmed by manual co-registration with CT-MRI images. Subdural electrodes were then projected on the 3D patient’s cortical model using *FreeSurfer*^59^. In case of simultaneous implantation of grids and/or strips with depth electrodes, the depth electrodes’ position was adjusted^60^. The type and number of implanted electrodes per patient are shown in Table 1.

### Identification of ictal onset and resection

All patients had clinical seizures whose onset and termination were marked by a board-certified epileptologist. We classified each electrode as “propagating” or “non-propagating” depending on whether it participated in seizure evolution (or not). Resected areas were identified by overlapping the pre-and post-operative MRIs. Accordingly, each electrode was further classified as “resected” or “non-resected” based on its overlap with these regions, and this classification was then confirmed with the post-surgical reports.

### FC estimation

After the removal of channels with artifacts, raw signals were band-pass (1–100 Hz) and notch filtered (60 Hz) with *Brainstorm*^58^. Filtered data were common average referenced to reduce common sources of noise from the signals^8,61–66^. On filtered data, we identified segments containing: (i) interictal activity with no or infrequent spikes (“No Spikes”); (ii) interictal activity with frequent spikes (“Spikes”); (iii) pre-ictal activity before the onset of a clinical seizure (“Pre-Ictal”); (iv) ictal activity during a clinical seizure (“Ictal”); and (v) post-ictal activity after the end of a clinical seizure (“Post-Ictal”). Artifact-free epochs of interictal activity were selected randomly, whereas pre- and post-ictal epochs were selected 1-min before and after the seizure. A total of 1-min of data (20 non-overlapping segments of 3 s duration each) was selected since it was found to generate stable FC networks^9,55,67^. For each segment, we computed three undirected FC measures (i.e. AEC, oAEC^68,69^ and PLV) across six physiologically relevant frequency bands (*delta*: 2–4 Hz; *theta*: 5–7 Hz; *alpha*: 8–12 Hz; *beta*: 15–29 Hz; *low-gamma*: 30–59 Hz); and *high-gamma*: 60–90 Hz). AEC estimates the amplitude correlation between two time series using the linear correlations of the envelopes of the filtered signals, which can detect signal coupling without phase coherence, even among different frequencies^9,70,71^. PLV measures the phase synchronization between two time series and is robust against fluctuations in signal amplitude^72^. Both methods have been extensively studied in epilepsy^9,71,73,74^ and are easily accessible in *Brainstorm*^58^. Each FC was then averaged across the 20 segments to obtain a unique averaged FC matrix, which contains the connectivity values between 0 (i.e., highest connectivity) and 1 (i.e., lowest connectivity) between pairs of electrodes, separately for epileptogenic states and frequency bands. Thus, we obtained three adjacency FC matrices (N×N) for each epileptogenic state and frequency band characterized by N *nodes* (i.e., iEEG electrodes) and N-1 *edges* (i.e., connectivity values between pairs of iEEG electrodes). To account for the decrease in FC values with increasing inter-regional distance^6,7,75^, we controlled for the distance between electrodes in patients with sEEG implantation or both sEEG and ECoG. Thus, we created a matrix containing the geometric (Euclidean) distance between all sEEG contact pairs of the same depth electrode and estimated the maximum distance among them^6^. We then normalized each depth electrode by the FC value of the contact having the highest Euclidean distance from the others. To quantify the degree of connectivity of each *node* in the network of each patient, we computed the nodal strength defined for each *node* as the average (median) of all the *edges* connected to that *node*. For each FC (i.e., AEC, oAEC, and PLV), we normalized the nodal strength of each electrode (i.e., sEEG and ECoG) by its maximum value respectively per patient, state, and band.

### Between connectivity and within connectivity analysis

To further investigate the FC in patients with good outcome who underwent sEEG implantation, we examined connectivity between epileptogenic and non-epileptogenic regions (“between connectivity”) and within each region (“within connectivity”). Specifically, we defined “between connectivity” as the median nodal strength across all pairs of sEEG contacts within a region compared to all other regions. Whereas “within connectivity” referred to the median nodal strength of only those sEEG contact pairs within a region (compared to other pairs within the same region). These analyses were conducted separately for epileptogenic (inside resection) and non-epileptogenic regions (outside resection), considering each FC measure, frequency band, and epileptogenic state. Furthermore, our analysis encompassed patients with sEEG implantation alone, as well as those with both ECoG and sEEG implantation. In cases where both electrode types were implanted, we analyzed exclusively the data from the sEEG electrodes.

### FSO and SSO analysis

For each patient, seizures were classified into two types based on a time-frequency analysis of their electrographic pattern before the seizure onset. We regarded as “FSO” a seizure with fast discharges in the beta or gamma band before the seizure onset; and as “SSO” a seizure with slow onset patterns involving theta or alpha band before the seizure onset. Patients showing both SSO and FSO were classified based on the highest number of seizures from either group. For each patient, we then used 1-min of recording from the same seizure type (either SSO or FSO); those patients who had ictal data with less than 1-min duration were excluded from the analysis. Finally, we analyzed AEC, oAEC, and PLV nodal strength estimated only on the ictal data.

### Identification of epileptogenic nodes

To assess whether resection of highly connected hubs (i.e., *nodes* with high nodal strength) can delineate the EZ, we examined whether these *nodes* were located inside (or outside) the EZ in good-outcome patients. Here, we assumed that in good-outcome patients, critical areas of the EZ were contained within the resection, whereas in poor-outcome patients, resection did not include critical EZ regions. We used AEC, oAEC, and PLV nodal strength of each *node* of good-outcome patients as a characteristic trademark for quantifying the ability to identify epileptogenic *nodes*. To identify the epileptogenicity of each node, we performed a ROC curve analysis with epileptogenicity thresholds that varied from 0 to 1 (with a step of 0.1). We defined as true positive (TP) a resected electrode correctly identified as epileptogenic, false positive (FP) a non-resected electrode identified as epileptogenic, false negative (FN) a resected electrode identified as non-epileptogenic, and true negative (TN) a non-resected electrode correctly identified as non-epileptogenic. Based on these assumptions, sensitivity [TP/(TP+FN)] and specificity [TN/(TN+FP)] were computed to obtain the ROC curve from which we estimated the area under the curve (AUC), defined as the ability of the classifier to accurately identify epileptogenic (inside resection) vs. non-epileptogenic (outside resection) *nodes*.

### Outcome prediction

To define if resected hubs (i.e. nodes with nodal strength above threshold) were able to delineate the EZ and predict outcome, we computed the overlap between hubs with resection and further assessed its association with outcome (*Fisher’s exact* test)^9^. For each FC (i.e., AEC, oAEC, PLV), the optimal connectivity threshold that defines these hubs was determined by extracting the maximum J among all ROC curves. Based on these results, we considered only those groups of hubs with nodal strength above their related thresholds. For each patient, we then estimated the percentage of overlap of hubs with resection by computing the ratio between only hubs located inside resection and the number of contacts clinically “resected”. We defined a zone as “clinically resected” when this percentage of overlap was higher than a defined threshold with values ranged from 0 to 100% (5% as a step). Thus, we considered TP a good outcome following resection, FP a poor outcome following resection, FN a good outcome following a missed resection, and TN a poor outcome following missed resection. Finally, we calculated the PPV [TP/(TP + FP)] and NPV [TN/(TN + FN)] values, and prediction accuracy ([TP + TN]/[TP + TN + FP + FN]). The best percentage of overlap of those hubs (with FC values above their connectivity thresholds) that need to be removed to achieve seizure freedom was obtained considering the maximum J, defined as J = [TP/(TP+FN)] + [TN/(TN+FP)] - 1.

### Statistical methods

We assessed the distribution of FC measures using the *Shapiro-Wilk normality* test and observed these data followed a non-normal distribution. To evaluate the ability of each FC measure to discriminate epileptogenic states, we compared the median nodal strength values of each patient across epileptogenic states (*Wilcoxon signed-rank* test), for each frequency band. Further, we used the *Mindboggle* atlas available in *Brainstorm*^58^ to classify electrodes as inside or outside the temporal lobe, separately for each patient. Based on this classification, we then compared the median nodal strength values of each patient across epileptogenic states separately for intra- and extra-temporal electrodes, as well as between these two types (*Wilcoxon signed-rank* test). For ictal data, we computed the median nodal strength between “propagating” and “not-propagating” electrodes, separately across all patients, and compared these values using *Wilcoxon signed-rank* test. The FDR method was used to correct significance for multiple comparisons. For each epileptogenic state and frequency band, we further compared the median nodal strength values between electrodes inside vs. outside resection, as well as inside vs. outside the clinically defined SOZ, respectively, for good- and poor-outcome patients (*Wilcoxon signed-rank* test). For the “between connectivity” and “within connectivity” analyses, we compared the nodal strength values of epileptogenic vs. non-epileptogenic regions using *Wilcoxon signed-rank* test. For each frequency band, we also compared the median nodal strength of FC measures for each patient with the age at surgery (*Spearman’s rank* correlation), separately for each state. We used *Wilcoxon rank-sum* test to compare the percentages of overlap of hubs with resection, as well as with the SOZ, between good- and poor-outcome patients, respectively. Statistical analysis was performed in MATLAB®2020a (The MathWorks, Inc); we considered a statistical significance for *p *< 0.05.

## Supplementary Information

Supplementary Information.

## Data Availability

Data supporting the results of this study are available from the corresponding author upon request.
