# Supplementary material for: Functional connectivity discriminates epileptogenic states and predicts surgical outcome in children with drug resistant epilepsy
Source: Sci Rep. 2023 Jun 14;13:9622. doi: 10.1038/s41598-023-36551-0 (PMC10267141; doi:10.1038/s41598-023-36551-0)
Supplement: Supplementary file 1 — Supplementary Information. [file 41598_2023_36551_MOESM1_ESM.docx]

**Supplementary Files**

**Functional connectivity discriminates epileptogenic states and predicts surgical outcome in children with drug resistant epilepsy**

**Sakar Rijal^1,2,†^, Ludovica Corona^1,2,†^, M. Scott Perry^1^, Eleonora Tamilia3, Joseph R. Madsen^4^, Scellig S.D. Stone^4^, Jeffrey Bolton^5^, Phillip L. Pearl^5^ & Christos Papadelis1,2,6***

^1^Jane and John Justin Institute for Mind Health Neurosciences Center, Cook Children's Health Care System, Fort Worth, TX, 76104, USA

^2^ Department of Bioengineering, The University of Texas at Arlington, Arlington, TX, 76010, USA

^3^ Fetal-Neonatal Neuroimaging and Developmental Science Center, Boston Children’s Hospital, Harvard Medical School, Boston, MA, 02115, USA

^4^ Division of Epilepsy Surgery, Department of Neurosurgery, Boston Children’s Hospital, Harvard Medical School, Boston, MA, 02115, USA

^5^ Division of Epilepsy and Clinical Neurophysiology, Department of Neurology, Boston Children’s Hospital, Harvard Medical School, Boston, MA, 02115, USA

^6^ School of Medicine, Texas Christian University, Fort Worth, TX, 76129, USA

**^†^** S. Rijal and L. Corona contributed equally.

Supplementary Figure S1

Supplementary Figure S2

Supplementary Figure S3

Supplementary Figure S4

Supplementary Figure S5

Supplementary Figure S6

Supplementary Figure S7

Supplementary Figure S8

Supplementary Table S1

Supplementary Table S2

For all supplementary Figures: AEC= Amplitude Envelope Correlation; oAEC = orthogonalized Amplitude Envelope Correlation; PLV= Phase Locking Value; FC = Functional Connectivity.

**Supplementary Fig. S1 Amplitude Envelope Correlation (AEC), orthogonalized AEC (oAEC), and Phase Locking Value (PLV) nodal strength for electrodes inside temporal lobes in physiologically relevant frequency bands.** AEC (*Top*), oAEC (*middle*), and PLV (*bottom*) nodal strength computed from electrodes inside temporal lobe for delta, theta, alpha, beta, low- and high- gamma frequency bands for different epileptogenic states. Each epileptogenic state is color-coded: interictal activity with no spikes (green), interictal activity with spikes (blue), pre-ictal activity before the onset of a clinical seizure (yellow), ictal activity during a clinical seizure (red), and post-ictal activity after the end of a clinical seizure (orange). Significant differences are marked with an asterisk (*) (*p* < 0.05, *Wilcoxon signed-rank* test). *P*-values are corrected for multiple comparisons using the false discovery rate method. In the box-plot diagrams, the horizontal line indicates the median value, lower and upper edges represent the 25^th^ and 75^th^ percentiles, whiskers extend to the minimum and maximum values (excluding outliers) and points outside the whiskers represent the outliers (i.e., values that are at least 1.5 times the interquartile range below the 25^th^ percentile or above the 75^th^ percentile).

**
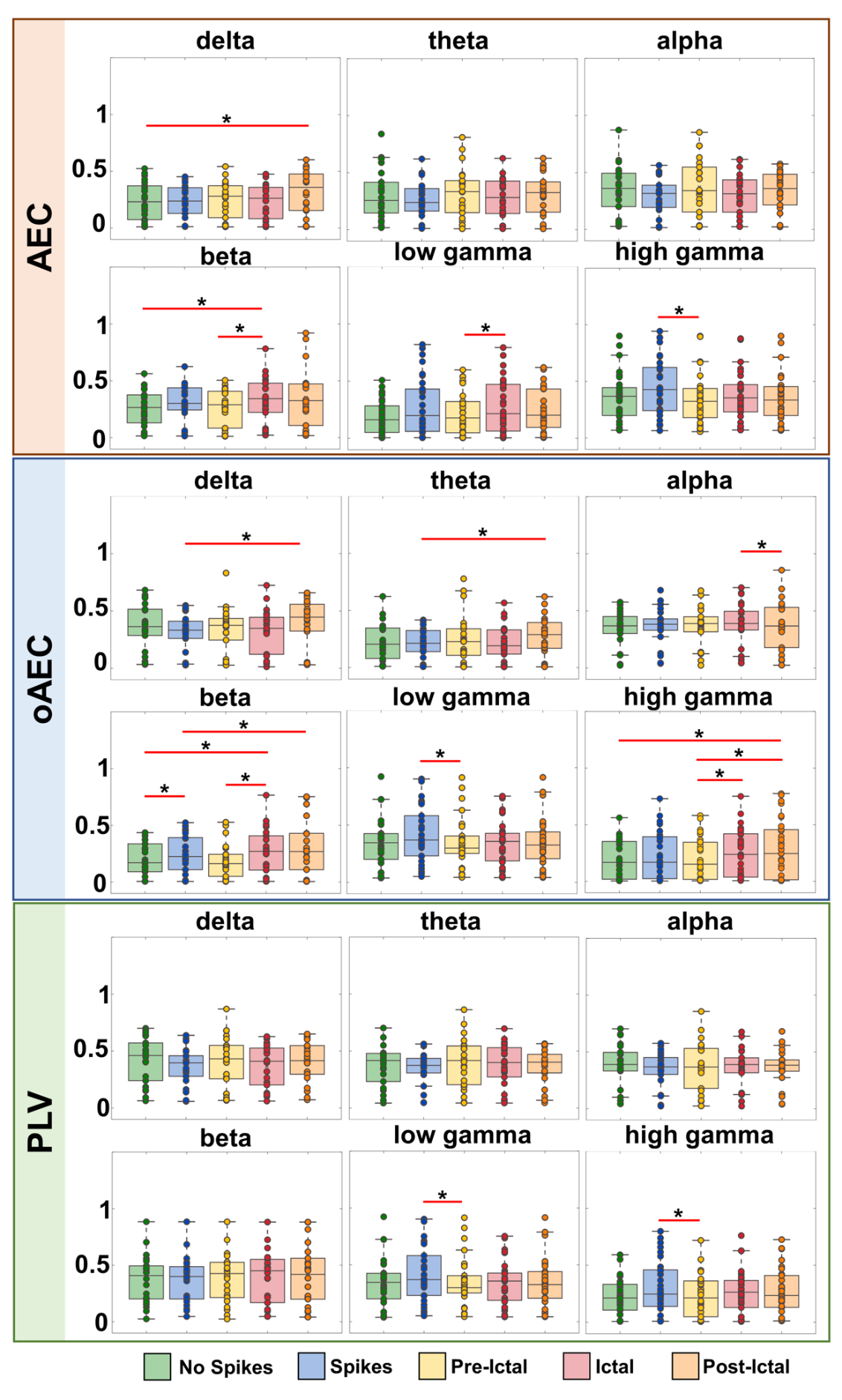
**

**Supplementary Fig. S2** **Amplitude Envelope Correlation (AEC), orthogonalized AEC (oAEC), and Phase Locking Value (PLV) nodal strength for electrodes inside the extra-temporal lobes in physiologically relevant frequency bands.** AEC (*Top*), oAEC (*middle*), and PLV (*bottom*) nodal strength computed from of electrodes outside temporal lobes for delta, theta, alpha, beta, low- and high- gamma frequency bands for different epileptogenic states. Each epileptogenic state is color-coded: interictal activity with no spikes (green), interictal activity with spikes (blue), pre-ictal activity before the onset of a clinical seizure (yellow), ictal activity during a clinical seizure (red), and post-ictal activity after the end of a clinical seizure (orange). Significant differences are marked with an asterisk (*) (*p* < 0.05, *Wilcoxon signed-rank* test). *P*-values are corrected for multiple comparisons using the false discovery rate method. In the box-plot diagrams, the horizontal line indicates the median value, lower and upper edges represent the 25^th^ and 75^th^ percentiles, whiskers extend to the minimum and maximum values (excluding outliers) and points outside the whiskers represent the outliers (i.e., values that are at least 1.5 times the interquartile range below the 25^th^ percentile or above the 75^th^ percentile).


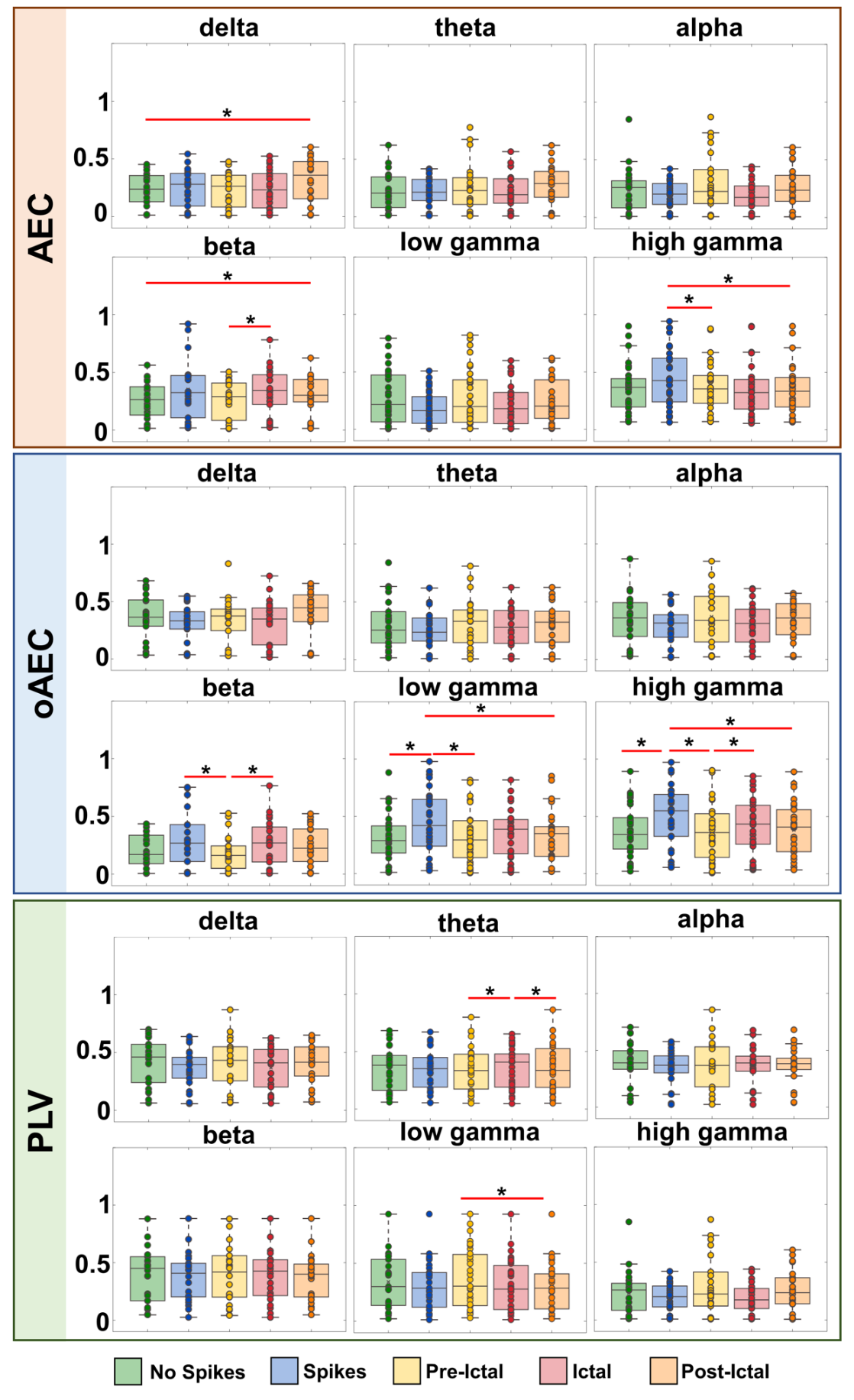


**Supplementary Fig. S3 Ictal nodal strength of electrodes involved *vs.* not involved in the seizure evolution for physiologically relevant frequency bands.** Amplitude Envelope Correlation (AEC) (*left*), orthogonalized AEC (oAEC) (*middle*), and Phase Locking Value (PLV) (*right*) nodal strength computed during ictal state from electrodes involved (blue-colored) and not-involved (orange-colored), separately across all patients for delta, theta, alpha, beta, low- and high-gamma frequency bands. In the box-plot diagrams, the horizontal line indicates the median value, the lower and upper edges represent the 25^th^ and 75^th^ percentiles, and the whiskers extend to the minimum and maximum values. *P*-values were obtained performing *Wilcoxon signed-rank* test.


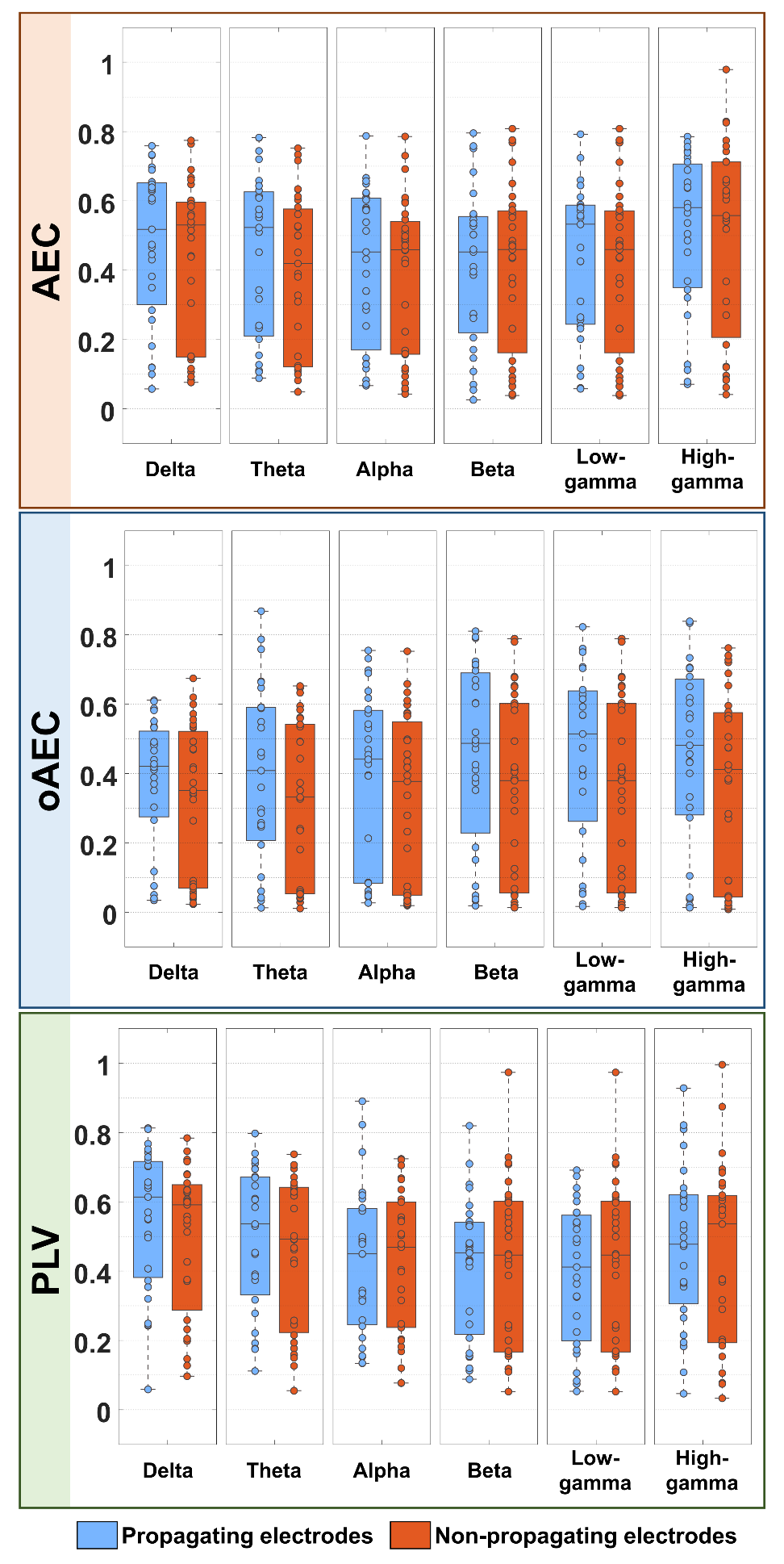


**Supplementary Fig. S4 Amplitude Envelope Correlation (AEC) nodal strength inside and outside resection for good- and poor-outcome patients in physiologically relevant frequency bands.** AEC nodal strength values computed from electrodes located inside vs. outside resection for all epileptogenic states in physiologically relevant frequency bands for patients with good (n=22, Engel I) and poor (n=9, Engel II-IV) surgical outcomes, respectively. Nodal strength values inside the resection are blue-colored; nodal strength values outside the resection are orange-colored. Significant differences are marked with an asterisk (*) (*p* < 0.05, *Wilcoxon signed-rank* test). In the box-plot diagrams, the horizontal line indicates the median value, the cross (×) sign indicates the mean, the lower and upper edges represent the 25^th^ and 75^th^ percentiles, and the whiskers extend to the minimum and maximum values.


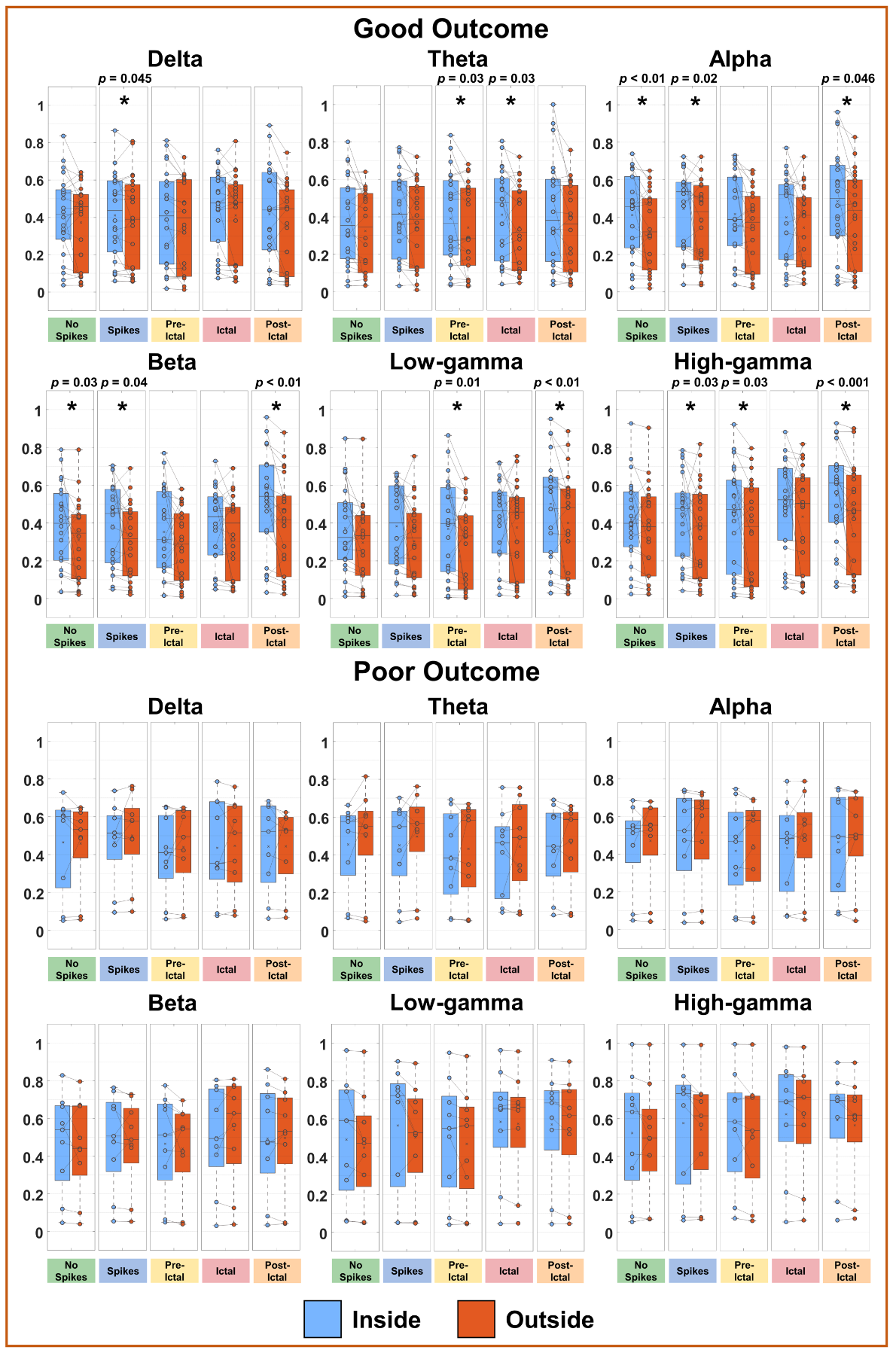


**Supplementary Fig. S5 Amplitude Envelope Correlation (AEC) nodal strength inside and outside the clinically defined Seizure Onset Zone (SOZ) for good- and poor-outcome patients in physiologically relevant frequency bands.** AEC nodal strength values computed from electrodes located inside and outside the SOZ for all epileptogenic states in physiologically relevant frequency bands for patients with good (n=22, Engel I) and poor (n=9, Engel II-IV) surgical outcomes, respectively. Nodal strength values inside the SOZ are blue-colored; nodal strength values outside the SOZ are orange-colored. Significant differences are marked with an asterisk (*) (*p* < 0.05, *Wilcoxon signed-rank* test). In the box-plot diagrams, the horizontal line indicates the median value, the cross (×) sign indicates the mean, the lower and upper edges represent the 25^th^ and 75^th^ percentiles, and the whiskers extend to the minimum and maximum values.


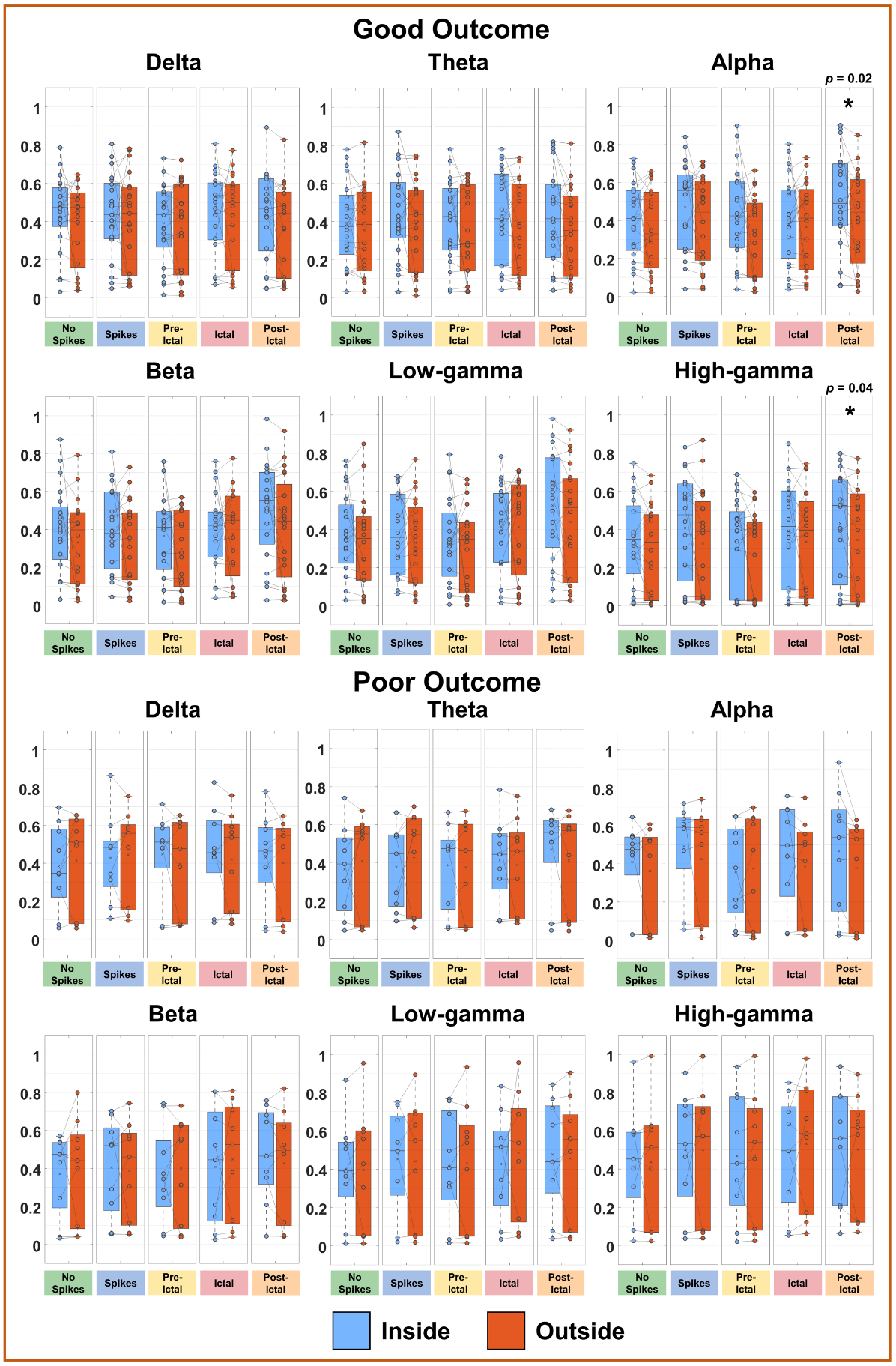


**Supplementary Fig. S6 Orthogonalized Amplitude Envelope Correlation (oAEC) nodal strength inside and outside the clinically defined Seizure Onset Zone (SOZ) for good- and poor-outcome patients in physiologically relevant frequency Bands.** oAEC nodal strength values computed from electrodes located inside and outside the SOZ for all epileptogenic states in physiologically relevant frequency bands for patients with good (n=22, Engel I) and poor (n=9, Engel II-IV) surgical outcomes, respectively. Nodal strength values inside the SOZ are blue-colored; nodal strength values outside the SOZ are orange-colored. Significant differences are marked with an asterisk (*) (*p* < 0.05, *Wilcoxon signed-rank* test). In the box-plot diagrams, the horizontal line indicates the median value, the cross (×) sign indicates the mean, the lower and upper edges represent the 25^th^ and 75^th^ percentiles, and the whiskers extend to the minimum and maximum values.

**
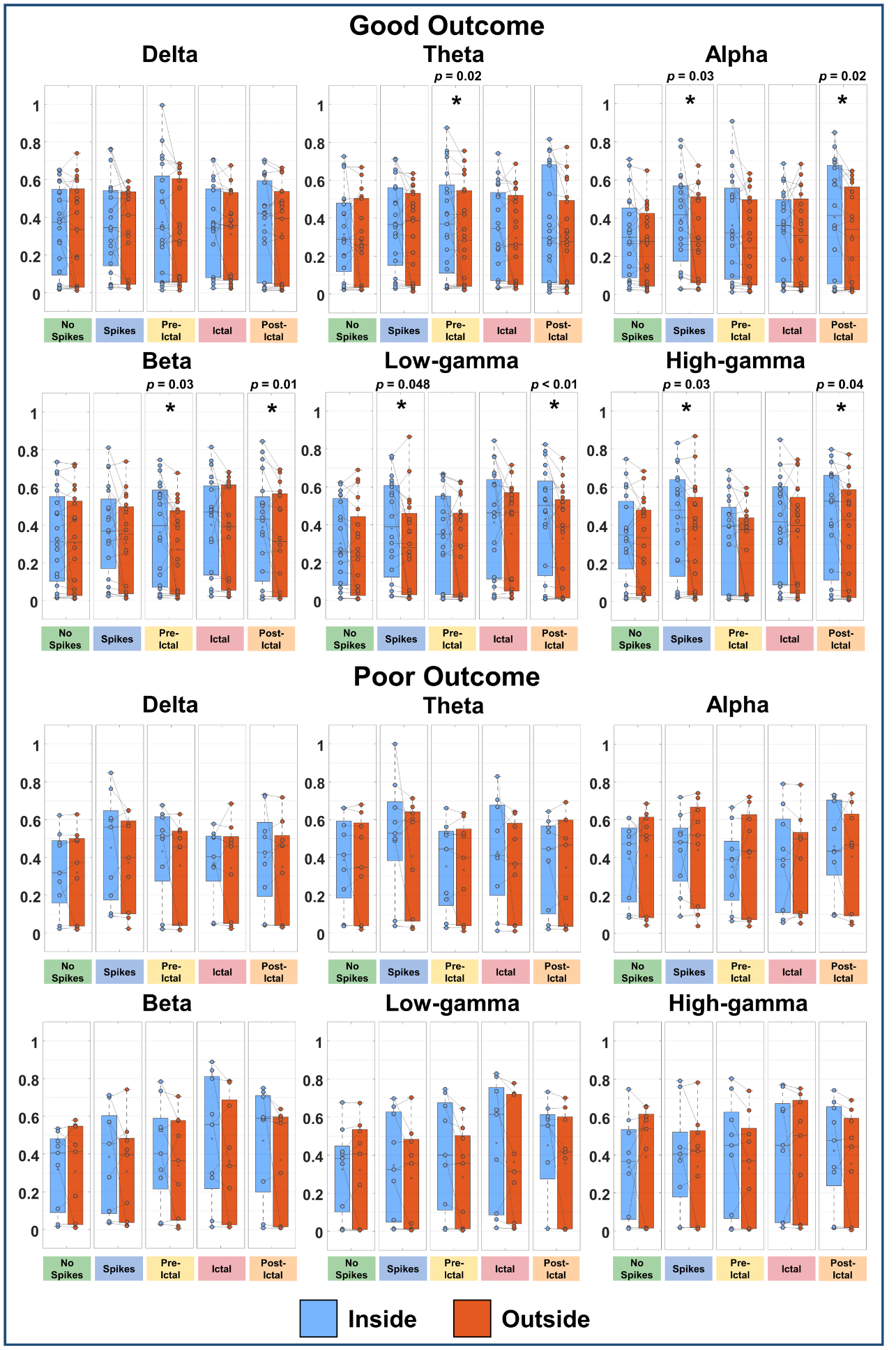
**

**Supplementary Fig. S7 Phase Locking Value (PLV) nodal strength inside and outside the clinically defined Seizure Onset Zone (SOZ) for good- and poor-outcome patients.** PLV nodal strength values computed from electrodes located inside and outside the SOZ for all epileptogenic states in physiologically relevant frequency bands for patients with good (n=22, Engel I) and poor (n=9, Engel II-IV) surgical outcomes, respectively. Nodal strength values inside the SOZ are blue-colored; nodal strength values outside the SOZ are orange-colored. Significant differences are marked with an asterisk (*) (*p* < 0.05, *Wilcoxon signed-rank* test). In the box-plot diagrams, the horizontal line indicates the median value, the cross (×) sign indicates the mean, the lower and upper edges represent the 25^th^ and 75^th^ percentiles, and the whiskers extend to the minimum and maximum values.


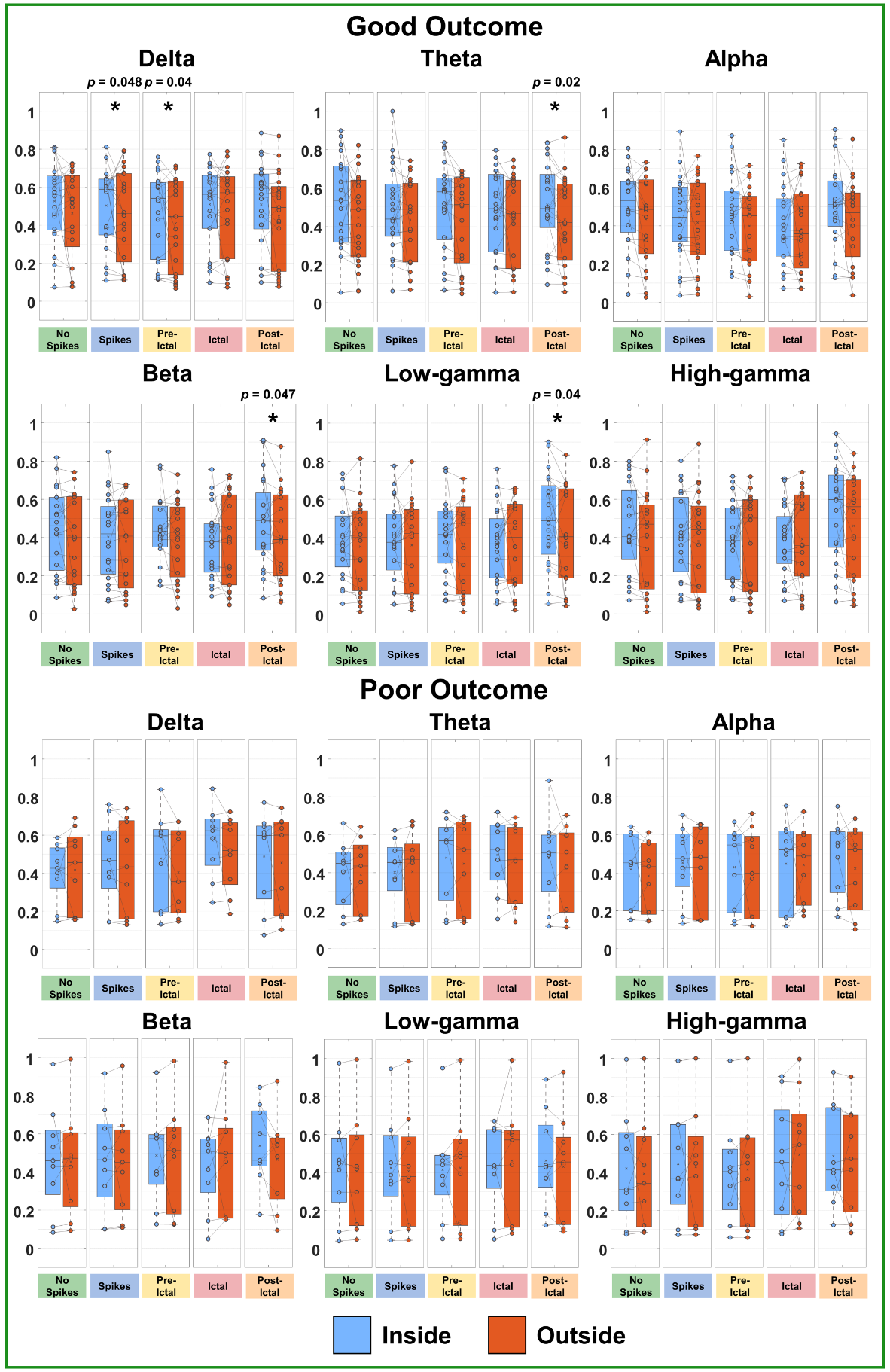


**Supplementary Fig. S8 Orthogonalized Amplitude Envelope Correlation (oAEC) values inside and outside epileptogenic regions for good-outcome patients having sEEG implantation.** *Top*: oAEC (“between connectivity”) computed in the interictal state with no spikes for a patient with good outcome (patient#1, Engel IA). “Between connectivity” represents the median nodal strength across all sEEG contact pairs within a region with all the other regions. For this patient, seizures originated from the posterior superior temporal gyrus showing increased oAEC for the theta, low- and high-gamma bands. Epileptogenic regions are red-labeled; non-epileptogenic regions are black-labeled. *Bottom panel*: oAEC (“between connectivity”) computed on interictal with no spikes (theta, low- and high-gamma bands), interictal spikes (low-gamma band), pre-ictal (delta band), and ictal (low-gamma band) states. oAEC values inside epileptogenic regions are blue-colored; oAEC values outside epileptogenic regions are orange-colored. Significant differences are marked with an asterisk (*) (*p* < 0.05, *Wilcoxon signed-rank* test). A = anterior; P = posterior; g = gyrus.


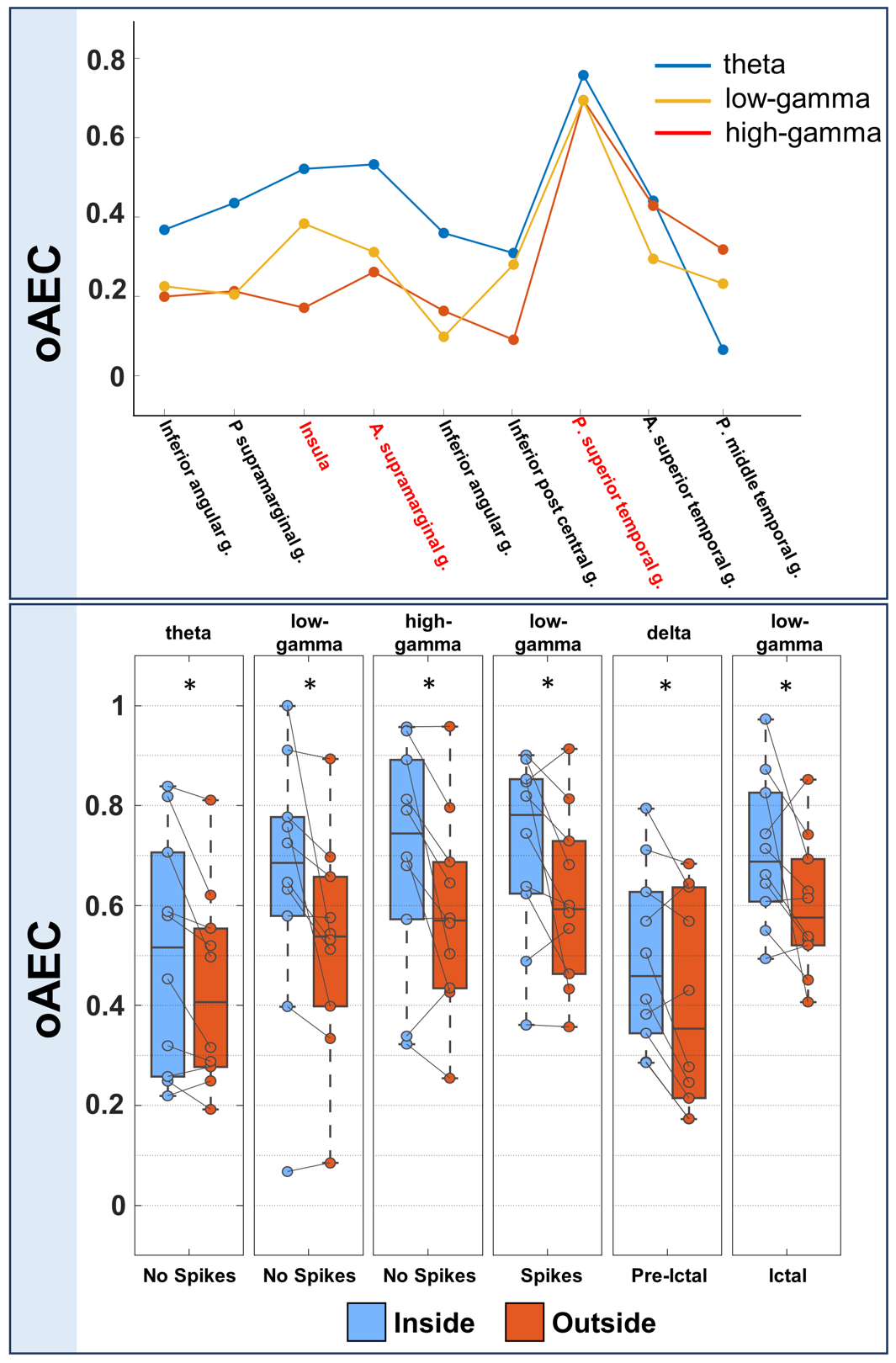


**Supplementary Table S1:**  **Results of nodal strength across states and bands for patients having only sEEG, ECoG, and both types of implantation.** Significant *p*-values obtained from the comparison between different epileptogenic states of the Amplitude Envelope Correlation (AEC), orthogonalized AEC (oAEC), and Phase Locking Value (PLV) nodal strength computed from for patients with only Electrocorticography (ECoG) implantation and patients with both types of implantation [ECoG and stereoelectroencephalography (sEEG)]. Statistically significant *p*-values (*Wilcoxon signed-rank* test) are displayed.

| **Amplitude Envelope Correlation** (**AEC**) | | |
| --- | --- | --- |
| **Patients with only ECoG implantation** | | |
| **Comparison between epileptogenic state** | **Frequency band** | ***p*-value** |
| Interictal with no spikes *vs.* Ictal | delta | 0.01 |
| Pre-Ictal *vs.* Ictal | delta | 0.04 |
| Post-Ictal *vs.* Ictal | delta | 0.03 |
| Interictal with no spikes *vs.* Interictal with spikes | theta | 0.02 |
| Interictal with no spikes *vs.* Ictal | theta | 0.01 |
| Interictal with no spikes *vs.* Interictal with spikes | alpha | 0.01 |
| Interictal with no spikes *vs.* Ictal | beta | 0.04 |
| Interictal with no spikes *vs.* Interictal with spikes | low-gamma | 0.02 |
| Interictal with no spikes *vs.* Ictal | low-gamma | 0.04 |
| Interictal with no spikes *vs.* Post-Ictal | low-gamma | 0.02 |
| Interictal with no spikes *vs.* Ictal | high-gamma | 0.03 |
| **Patients with both ECoG and sEEG implantation** | | |
| **Comparison between epileptogenic state** | **Frequency band** | ***p*-value** |
| Pre-Ictal *vs.* Ictal | delta | 0.03 |
| Post-Ictal *vs.* Ictal | delta | 0.02 |
| Pre-Ictal *vs.* Ictal | theta | 0.04 |
| Interictal with no spikes *vs.* Ictal | beta | 0.01 |
| Pre-Ictal *vs.* Ictal | low-gamma | 0.02 |
| Pre-Ictal *vs.* Post-Ictal | high-gamma | 0.01 |
| **Orthogonalized Amplitude Envelope Correlation** (**oAEC**) | | |
| **Patients with only ECoG implantation** | | |
| **Comparison between epileptogenic state** | **Frequency band** | ***p*-value** |
| Interictal with no spikes *vs.* Ictal | delta | 0.04 |
| Interictal with spikes *vs.* Ictal | delta | 0.03 |
| Pre-Ictal *vs.* Ictal | delta | 0.03 |
| Post-ictal *vs.* Ictal | delta | 0.01 |
| Interictal with no spikes *vs.* Ictal | theta | 0.03 |
| Post-ictal *vs.* Ictal | theta | 0.02 |
| Interictal with no spikes *vs.* Ictal | alpha | 0.03 |
| Interictal with no spikes *vs.* Interictal with spikes | beta | 0.03 |
| Interictal with no spikes *vs.* Ictal | beta | 0.02 |
| Pre-Ictal *vs.* Ictal | beta | 0.04 |
| Pre-Ictal *vs.* Interictal with spikes | beta | 0.02 |
| Interictal with no spikes *vs.* Ictal | low-gamma | 0.02 |
| Pre-Ictal *vs.* Ictal | low-gamma | 0.02 |
| Interictal with no spikes *vs.* Ictal | high-gamma | 0.01 |
| Pre-Ictal *vs.* Interictal with spikes | high-gamma | 0.03 |
| Pre-Ictal *vs.* Ictal | high-gamma | 0.04 |
| **Patients with both ECoG and sEEG implantation** | | |
| **Comparison between epileptogenic state** | **Frequency band** | ***p*-value** |
| Interictal with no spikes *vs.* Interictal with spikes | delta | 0.01 |
| Pre-Ictal *vs.* Post-Ictal | delta | 0.01 |
| Ictal *vs.* Post-Ictal | delta | 0.02 |
| Interictal with no spikes *vs.* Interictal with spikes | theta | 0.02 |
| Pre-Ictal *vs.* Post-Ictal | theta | 0.03 |
| Ictal *vs.* Post-Ictal | theta | 0.01 |
| Interictal with no spikes *vs.* Interictal with spikes | alpha | 0.03 |
| Interictal with no spikes *vs.* Interictal with spikes | low-gamma | 0.04 |
| Pre-Ictal *vs.* Ictal | low-gamma | 0.02 |
| **Phase Locking Value** (**PLV**) | | |
| **Patients with only ECoG implantation** | | |
| **Comparison between epileptogenic state** | **Frequency band** | ***p*-value** |
| Interictal with no spikes *vs.* Interictal with spikes | alpha | 0.01 |
| **Patients with both ECoG and sEEG implantation** | | |
| **Comparison between epileptogenic state** | **Frequency band** | ***p*-value** |
| Pre-Ictal *vs.* Ictal | delta | 0.02 |
| Interictal with spikes *vs.* Interictal with no spikes | alpha | 0.03 |

**Supplementary Table S2: Results of nodal strength compared between patients with only stereoelectroencephalography (sEEG) and patients with only electrocorticography (ECoG) implantations.** Significant *p*-values obtained from the comparison of the Amplitude Envelope Correlation (AEC), orthogonalized AEC (oAEC), and Phase Locking Value (PLV) nodal strength between patients with only Electrocorticography (ECoG) implantation and patients with both types of implantation [ECoG and sEEG] across epileptogenic states. *P*-values are obtained performing Wilcoxon rank-sum test and corrected for multiple comparisons using the False discovery rate method. Empty cells represent the non-statistical significance (*p*>0.05).

| **Nodal strength of sEEG vs. ECoG** | | | | | | |
| --- | --- | --- | --- | --- | --- | --- |
| **Epileptogenic states** | **Amplitude Envelope Correlation** (**AEC**) | | | | | |
|  | delta | theta | alpha | beta | low-gamma | high-gamma |
| Interictal with no spikes |  |  |  |  |  | 0.01 |
| Interictal with spikes |  |  |  |  |  |  |
| Pre-Ictal |  |  |  |  |  |  |
| Ictal |  |  |  |  |  |  |
| Post-Ictal |  |  |  |  |  |  |
|  | **Orthogonalized Amplitude Envelope Correlation** (**oAEC**) | | | | | |
| Interictal with no spikes |  | 0.01 |  |  |  |  |
| Interictal with spikes |  | 0.007 |  |  |  |  |
| Pre-Ictal |  | 0.005 |  |  |  |  |
| Ictal |  | 0.003 |  |  |  |  |
| Post-Ictal | 0.03 | 0.007 |  |  | 0.03 |  |
|  | **Phase Locking Value** (**PLV**) | | | | | |
| Interictal with no spikes |  | 0.02 | 0.005 | 0.015 |  |  |
| Interictal with spikes |  |  | 0.01 | 0.001 |  |  |
| Pre-Ictal |  |  | 0.01 | 0.02 |  |  |
| Ictal | 0.01 |  | 0.005 | 0.01 |  | 0.03 |
| Post-Ictal |  |  | 0.007 | 0.005 |  | 0.02 |
